# Molecular characterization, phylogenetic and variation analyses of SARS-CoV-2 strains in Turkey

**DOI:** 10.2217/fmb-2021-0118

**Published:** 2021-10-07

**Authors:** Murat Karamese, Didem Ozgur, Emin E Tutuncu

**Affiliations:** ^1^Department of Medical Microbiology, Kafkas University, Kars, 36100, Turkey; ^2^Department of Infectious Diseases & Clinical Microbiology, Kafkas University, Kars, 36100, Turkey

**Keywords:** complete genome sequencing, COVID-19, phylogenetic analysis, SARS-CoV-2, SNP analysis

## Abstract

**Aims:** We present the sequence and single-nucleotide polymorphism (SNP) analysis for 47 complete genomes for SARS-CoV-2 isolates on Turkish patients. **Methods:** The Illumina MiSeq platform was used for sequencing the libraries. The SNPs were detected by using Genome Analysis Toolkit – HaplotypeCaller v.3.8.0 and were inspected on GenomeBrowse v2.1.2. **Results:** All viral genome sequences of our isolates were located in lineage B under the different clusters, such as B.1 (n = 3), B.1.1 (n = 28) and B.1.9 (n = 16). According to the Global Initiative on Sharing All Influenza Data nomenclature, all of our complete genomes were placed in G, GR and GH clades. In our study, 549 total and 53 unique SNPs were detected. **Conclusion:** The results indicate that the SARS-CoV-2 sequences of our isolates have great similarity with all Turkish and European sequences.

A new virus-associated disease, COVID-19 by SARS-CoV-2, was first declared in late December 2019 in Wuhan, China [1]. As of this writing, there have been more than 173 million confirmed cases and more than 3.7 million deaths reported worldwide [2]. The etiological agent of this disease, SARS‐CoV‐2, has an ssRNA (single stranded) genome and its length is approximately 29,890 base pairs according to the data obtained from NCBI GenBank (GenBank NC_045512.2) [3]. After the confirmation of human-to-human transmission and extensive global spread, WHO declared COVID-19 as a pandemic on 11 March 2020 [4].

Understanding of the transmission patterns and evolution of SARS-CoV-2 virus is crucial and necessary for creating better drug and vaccine designs for disease control and prevention [4–6]. The analysis of genetic sequence data from a pathogen is known as an important tool in infectious disease epidemiology [7,8]. There are nearly 1.9 million pieces of SARS-CoV-2 virus data available on the database of Global Initiative on Sharing All Influenza Data (GISAID) [9]. The availability of these genomic data has been helping hundreds of researchers to analyze the genomic diversity of SARS-CoV-2 virus. First, Rambaut *et al.* [8] have defined the lineage A and B (both of them include sublineages such as A.1, B.1 and A.1.1) to help with tracking and understanding the patterns and determinants of the global spread of SARS-CoV-2. Then, on 4 July 2020, GISAID published the new clade and lineage nomenclature aids in genomic epidemiology studies of SARS-CoV-2 viruses. They developed a nomenclature system based on marker mutations in phylogenetic groups and named them S, L, V, G, GH and GR [10].

Next-generation sequencing (NGS) is a term that describes a DNA-sequencing technology. This technology is a reliable and powerful high-throughput tool and can be used to perform a sequencing of the whole genomes or constrained to specific areas of interest. The most important advantages of the NGS technique are capturing a broader spectrum of mutations than Sanger sequencing and being more sensitive to detection of mosaic mutations [11,12].

In this study, we performed NGS analysis for 47 complete genomes for SARS-CoV-2 isolates in Turkish patients. To identify their genetic similarity, phylogenetic analysis was performed by comparing the worldwide SARS-CoV-2 sequences, selected from GISAID, with the complete genomes from Turkish isolates. In addition, we focused on the variation analysis to show the mutations on SARS-CoV-2 genomes.

The obtained data were one of the first data both in the country and all around the world that contained 47 full-genome sequences of SARS-CoV-2. Similar studies with more data should be performed to fight against COVID-19. Additionally, the scientific field needs new similar data until an effective vaccine or drug is developed.

## Methods

This study was approved by both the Republic of Turkey Ministry of Health COVID-19 Scientific Research Evaluation Commission (approval date: 02/05/2020; number: 2020-05-02T16_13_50) and the Local Ethics Committee of Kafkas University Faculty of Medicine (approval date: 06/05/2020 number: 80576354-050-99/130).

Of the patients who tested positive for SARS-CoV-2, the 63 samples with the highest viral loads (assessed by real-time PCR by Biospeedy SARS-CoV-2 qPCR Kit targeting the RdRp gene) were selected for NGS-based assays. All samples were the first sample of patients (first positivity). After NGS analysis, we used 47 of those samples for complete genome sequencing and variation analyses.

The library preparation was performed using CleanPlex^®^ SARS-CoV-2 Panel (Paragon Genomics Inc, IA, USA) with isolated RNA samples following the manufacturer instructions. Then, Illumina MiSeq (Illumina Inc., CA, USA) platform with a 2× 150-cycle kit was used for sequencing the libraries. The quality of the raw data was examined by FastQC v.0.11.5, and low-quality bases and primers were trimmed using Trimmomatic (version 0.32). The raw reads were aligned to the known SARS-CoV-2 genome (GenBank Accession: MN908947.3) using the Burrows-Wheeler aligner (version 0.7.1). The consensus sequences were aligned via multiple sequence alignment using the MAFFT v7.450 tool. The phylogenetic tree was constructed using Phylip v.3.6 with a neighbor-joining and composite likelihood method and 100 bootstrap replications. For phylogenetic analysis of our data, the GISAID database was used to collect SARS-CoV-2 complete genomes of different patients from all around the world (i.e., Belgium, Bosnian-Herzegovina, Czech Republic, Croatia, Cyprus, Finland, France, Germany, Hungary, Italy, Poland, Portugal, Spain, Russia, Australia, USA, Mexico, Costa Rica, Canada, Austria, Thailand, China, Kuwait, Senegal, Egypt, Japan, Chile, Nigeria, Algeria, Brazil, Ecuador, Mongolia, Colombia, India, Indonesia, Malaysia, Bangladesh, Kazakhstan and Turkey). Only complete genomes (28,000–30,000 base pairs) were analyzed. The SNPs were detected by using Genome Analysis Toolkit – HaplotypeCaller (GATK) v.3.8.0 and were inspected on GenomeBrowse v2.1.2 (GoldenHelix). The SNPs with low quality and a low variant fraction (%<60) were removed. The filtered SNPs and reference SARS-CoV-2 genome were used to generate the consensus sequence using bcftools v1.9.

## Results

**Figure 1.**
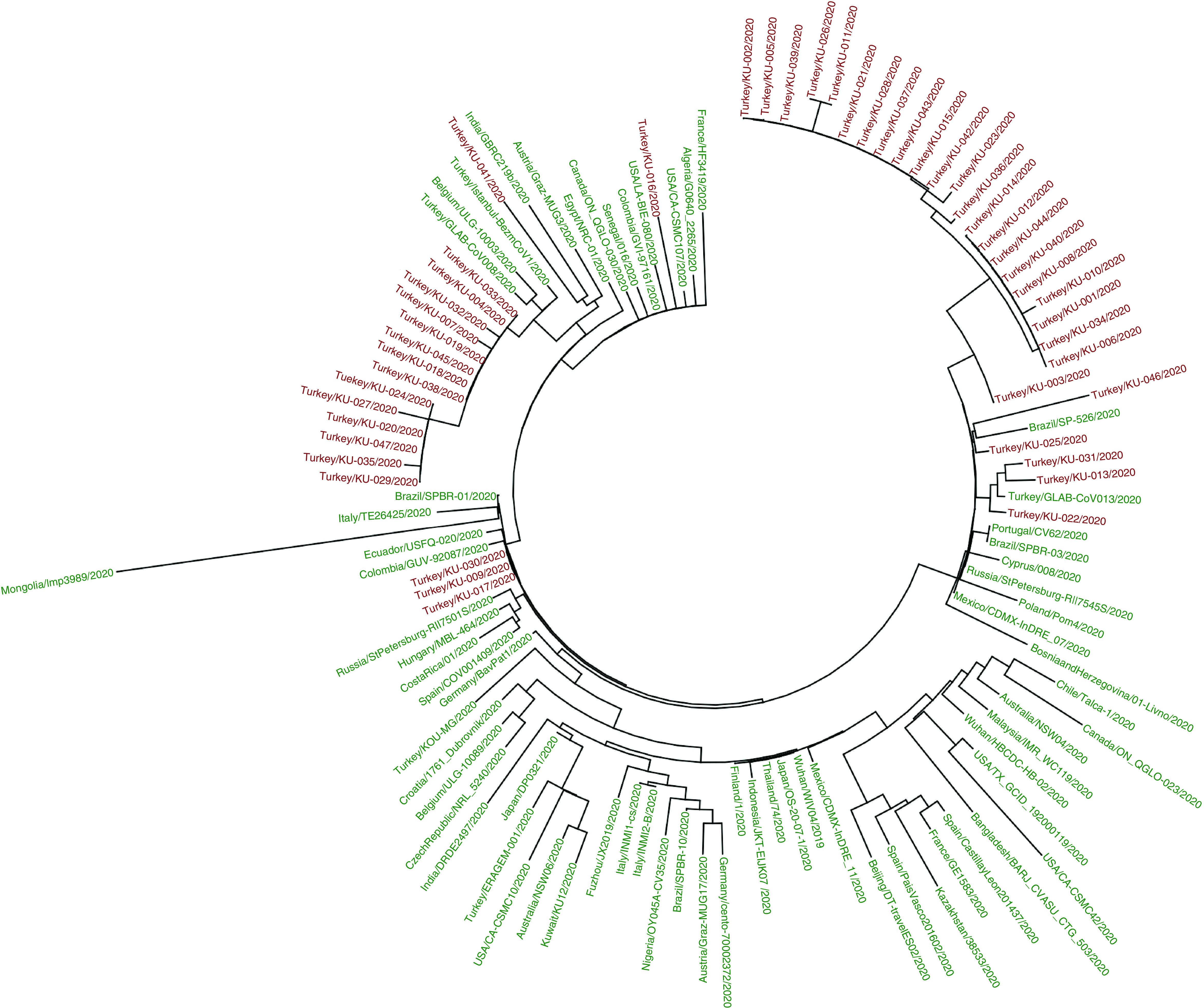
Subtree showing the SARS-CoV-2 strains containing also at least one strain in different countries. Our viruses are shown in red font color.

A database of 68 complete genomes of SARS-CoV-2 strains belonging to different countries randomly selected from the GISAID database were compared with our isolations. The reference SARS-CoV-2 strain, which was rooted from Wuhan (NCBI GenBank, NC_045512) was also selected for comparison. A total of 115 SARS-CoV-2 genomes were placed in the phylogenetic tree. The maximum likelihood phylogenetic tree in Figure 1 shows a main lineage including several sublineages. All viral genome sequences of our isolates were located in lineage B under the different clusters, such as B.1 (n = 3), B.1.1 (n = 28) and B.1.9 (n = 16). According to the GISAID nomenclature, all of our complete genomes were placed in G, GR and GH clades (Figure 1 & Supplementary Table 1).

In our study, 549 total and 53 unique SNPs were detected as shown in Supplementary Table 2. All 47 genomes exhibited different kinds of SNPs. The distinct SNPs consist of 274 missense, 225 synonymous and 50 noncoding alleles (Supplementary Table 2). The most common detected SNPs were c.1-25G>T (5′UTR), c.2772C>T (ORF1ab), c.14143C>T (ORF1ab), c.1841A>G (S) in all our genomes (n = 47), c.608G>A (N), c.609G>A (N), c.610G>C (N) in 28 samples, and c.2700G>T (S), c.310G>T (ORF7a) in 23 samples. Additionally, 31 SNPs were detected only once. Two hundred ninety-one (53%) SNPs were detected in ORF1ab, which is the longest ORF consisting of approximately 70% of the whole genome. The ORF1ab is cleaved into 16 nonstructural proteins (nsp). Among those, nsp3 and nsp2 had more SNPs in our study (n = 100 and n = 27, respectively). However, all noncoding mutations were detected in the 5′UTR region. All genomes had c.1-25C>T nucleotide variation, but only two genomes had additional noncoding mutations: c.1-56G>T and c.1-77G>T. In terms of base changing, the one most commonly detected (96%) was C>T. The detailed data about coding and noncoding mutations in our isolates is shown in Supplementary Table 2.

## Discussion

The analysis of complete genome sequencing of new viruses is an important tool for epidemiology of infectious diseases, updating diagnostics and assessing viral evolution [7,8]. Complete genome data of a virus makes visible some epidemiological parameters including doubling time of an outbreak, reconstruction of transmission routes, and the identification of possible sources and animal reservoirs. It can also help the study of drug and vaccine design. In our study, we described 47 SARS-CoV-2 genomes and compared them with a dataset of 68 available SARS-CoV-2 genomes from different countries obtained from GISAID [9].

After the phylogenetic analysis, it was shown that all of our SARS-CoV-2 genomes are similar to the European strains. According to GISAID lineage, 47 of 47 of our SARS-CoV-2 genomes are in lineage B, which mostly consists of European strains. According to the marker mutations, those were placed in G, GR and GH clades. Moreover, most of European isolates were placed in G, GR and GH clades in GISAID [9].

In current literature, there are some studies that investigate the first isolates of their country to find the origin of those [3,13–17]. One study contains two complete genomes of a Chinese patient visiting Rome and an Italian patient, reporting that the Italian patient’s sequence clustered with European sequences and the Chinese patient’s sequence clustered with the Wuhan sequence (NC_045512) [18]. The same researchers compared the two genomes regarding concurrent evolution and accumulation of mutations and reported that four mutations were detected in the Italian sequence. Another study reported that because of the variations, the first isolates of India did not highly identify with the Wuhan sequence (NC_045512) [17]. Moreover, Bal *et al.* reported that the first three French sequences were located in the European clade instead of the reference Wuhan clade because of the three-nucleotide deletion in Orf1a at positions 1607–1609 [13]. However, some European isolates that were generally the first isolates of a related country belong to non-European clades. A German sequence was evaluated in a study performed by Zehender *et al.* [19] and reported that it belonged to lineage A because of traveling to Shanghai between 20 and 24 January.

So far, there is a total of 138 SARS-Cov-2 complete genome sequence data except for our 47 isolates in the GISAID database from Turkey [9]. It can be seen that all of those genomes belong to lineage B, including the first case (lineage B.4) in Turkey. All sequences (n = 47) belong to lineage B, similar to all other Turkish sequences. However, the first cases in Turkey belong to the L clade according to GISAID classification, whereas ours belong to G, GR and GH clades.

However, the length of the SARS-CoV-2 genome is approximately 30 Kb including 5′UTR and 3′UTR noncoding sequences, *Orf1ab*, *S*, *Orf3a*, *E*, *M*, *Orf6a*, *Orf7a*, *Orf7b*, *Orf8*, *N* and *Orf10* genes. In this study, we aim to detect the variations of our SARS-CoV-2 isolates.

According to the current literature, ORF genes have a crucial role in COVID-19 [20]. So, in our study, 549 total and 53 unique SNPs were detected from 47 SARS-CoV-2 isolates (Supplementary Table 2). A study performed by Khailany *et al.* [21] reported that 156 total and 116 unique SNPs were found in 95 SARS-CoV-2 isolates. The same study also pointed out that the most frequently observed base changes were C>T, the same as our study.

The most observed variations are found in positions 3036, 11083 and 13402 belonging to the *Orf1ab* gene; 28854 belonging to the *N* gene; 21707 and 21575 belonging to the *S* gene; and 28077 and 28144 belonging to the *Orf8* gene [4,17,22,23]. Our variations in related positions are the same as those. We mostly detected variations in positions 3037 and 11083 in the *Orf1ab* gene; 28166 in the *Orf8* gene; 28854 in the *N* gene and 24262 in the *S* gene. According to the global frequency of variations, most of our SNPs are novel; however, the studies on this issue are ongoing, and more detailed information will be presented in the near future. *Orf1ab* is the longest and most important gene among coronaviruses [20]. Most of the scientists detected variations in the *Orf1ab* gene. Thus, the mutations in this region may be significant concerning clinical features.

## Conclusion

The results of the present study indicated that the SARS-CoV-2 sequences of our isolates have great similarity with all Turkish and European sequences. Further studies should be performed for better comparison of strains, after more complete genome sequences are released; however, these data may be useful to understand the dynamics of virus spread and may help further vaccine and treatment studies. The increase of SARS-CoV-2 cases all over the world is giving more genomes that may present some visibility of populace structure. This study showed the common and new variations in SARS-CoV-2 isolates. The fight against COVID-19 will last a long time until an effective vaccine or drug is developed. However, we believe that collecting and sharing any data about SARS-CoV-2 virus and COVID-19 will be effective and may help the related studies. At that point, we should carry on with detecting new variations.

## Study limitations

Our study has some limitations: the number of national genomes available at the time of analysis and the number of detected variations. Additionally, not using a more robust methodology, such as maximum likelihood, is another limitation of the study. These limitations should be eliminated for further studies.

**Summary points**

- To understand the transmission patterns and evolution of SARS-CoV-2, it is crucial and necessary to create better drug and vaccine designs for disease control and prevention. The analysis of genetic sequence data from a pathogen is known as an important tool in infectious disease epidemiology.
- Forty-seven positive samples were used for complete genome sequencing and variation analyses. A database of 68 complete genomes of SARS-CoV-2 strains belonging to different countries randomly selected from the Global Initiative on Sharing All Influenza Data database were compared with our isolations.
- The results of present study indicated that the SARS-CoV-2 sequences of our isolates have great similarity with all Turkish and European sequences.
- The fight against COVID-19 will last a long time until an effective vaccine or drug is developed. However, we believe that collecting and sharing any data about the SARS-CoV-2 virus and COVID-19 will be effective and may help the related studies. At that point, we should carry on detecting the new variations.
