# Supplementary material for: Molecular characterization, phylogenetic and variation analyses of SARS-CoV-2 strains in Turkey
Source: Future Microbiol. 2021 Oct 7:10.2217/fmb-2021-0118. doi: 10.2217/fmb-2021-0118 (PMC8507978; doi:10.2217/fmb-2021-0118)
Supplement: Supplementary file 1 [file Supplementary_Table_1.docx]

**Supplementary Table 1:** The accession numbers obtained from GenBank and GISAID database ad GISAID clades of our isolates

| **GenBank Name** | **GenBank Accession Number** | **GISAID Name** | **GISAID Accession Number** | **GISAID Clade** |
| --- | --- | --- | --- | --- |
| Kafkas-SARSCoV2-001 | MT786327 | hCoV-19/Turkey/KU-001/2020 | EPI_ISL_495411 | B.1.1 (GR) |
| Kafkas-SARSCoV2-002 | MT786861 | hCoV-19/Turkey/KU-002/2020 | EPI_ISL_495412 | B.1.1 (GR) |
| Kafkas-SARSCoV2-003 | MT786865 | hCoV-19/Turkey/KU-003/2020 | EPI_ISL_495413 | B.1.1 (GR) |
| Kafkas-SARSCoV2-004 | MT786329 | hCoV-19/Turkey/KU-004/2020 | EPI_ISL_495414 | B.1.9 (GH) |
| Kafkas-SARSCoV2-005 | MT786797 | hCoV-19/Turkey/KU-005/2020 | EPI_ISL_495415 | B.1.1 (GR) |
| Kafkas-SARSCoV2-006 | MT786331 | hCoV-19/Turkey/KU-006/2020 | EPI_ISL_495416 | B.1.1 (GR) |
| Kafkas-SARSCoV2-007 | MT786334 | hCoV-19/Turkey/KU-007/2020 | EPI_ISL_495417 | B.1.9 (GH) |
| Kafkas-SARSCoV2-008 | MT786859 | hCoV-19/Turkey/KU-008/2020 | EPI_ISL_495418 | B.1.1 (GR) |
| Kafkas-SARSCoV2-009 | MT786860 | hCoV-19/Turkey/KU-009/2020 | EPI_ISL_495419 | B.1 (G) |
| Kafkas-SARSCoV2-010 | MT786335 | hCoV-19/Turkey/KU-010/2020 | EPI_ISL_495420 | B.1.1 (GR) |
| Kafkas-SARSCoV2-011 | MT786868 | hCoV-19/Turkey/KU-011/2020 | EPI_ISL_495421 | B.1.1 (GR) |
| Kafkas-SARSCoV2-012 | MT787647 | hCoV-19/Turkey/KU-012/2020 | EPI_ISL_495422 | B.1.1 (GR) |
| Kafkas-SARSCoV2-013 | MT787564 | hCoV-19/Turkey/KU-013/2020 | EPI_ISL_495423 | B.1.1 (GR) |
| Kafkas-SARSCoV2-014 | MT786866 | hCoV-19/Turkey/KU-014/2020 | EPI_ISL_495424 | B.1.1 (GR) |
| Kafkas-SARSCoV2-015 | MT787648 | hCoV-19/Turkey/KU-015/2020 | EPI_ISL_495425 | B.1.1 (GR) |
| Kafkas-SARSCoV2-016 | MT787650 | hCoV-19/Turkey/KU-016/2020 | EPI_ISL_495426 | B.1 (GH) |
| Kafkas-SARSCoV2-017 | MT787578 | hCoV-19/Turkey/KU-017/2020 | EPI_ISL_495427 | B.1 (G) |
| Kafkas-SARSCoV2-018 | MT787645 | hCoV-19/Turkey/KU-018/2020 | EPI_ISL_495428 | B.1.9 (GH) |
| Kafkas-SARSCoV2-019 | MT787577 | hCoV-19/Turkey/KU-019/2020 | EPI_ISL_495429 | B.1.9 (GH) |
| Kafkas-SARSCoV2-020 | MT787646 | hCoV-19/Turkey/KU-020/2020 | EPI_ISL_495430 | B.1.9 (GH) |
| Kafkas-SARSCoV2-021 | MT787582 | hCoV-19/Turkey/KU-021/2020 | EPI_ISL_495431 | B.1.1 (GR) |
| Kafkas-SARSCoV2-022 | MT787581 | hCoV-19/Turkey/KU-022/2020 | EPI_ISL_495432 | B.1.1 (GR) |
| Kafkas-SARSCoV2-023 | MT786336 | hCoV-19/Turkey/KU-023/2020 | EPI_ISL_495433 | B.1.1 (GR) |
| Kafkas-SARSCoV2-024 | MT786337 | hCoV-19/Turkey/KU-024/2020 | EPI_ISL_495434 | B.1.9 (GH) |
| Kafkas-SARSCoV2-025 | MT787580 | hCoV-19/Turkey/KU-025/2020 | EPI_ISL_495435 | B.1.1 (GR) |
| Kafkas-SARSCoV2-026 | MT789689 | hCoV-19/Turkey/KU-026/2020 | EPI_ISL_495436 | B.1.1 (GR) |
| Kafkas-SARSCoV2-027 | MT787500 | hCoV-19/Turkey/KU-027/2020 | EPI_ISL_495437 | B.1.9 (GH) |
| Kafkas-SARSCoV2-028 | MT787749 | hCoV-19/Turkey/KU-028/2020 | EPI_ISL_495438 | B.1.1 (GR) |
| Kafkas-SARSCoV2-029 | MT787745 | hCoV-19/Turkey/KU-029/2020 | EPI_ISL_495439 | B.1.9 (GH) |
| Kafkas-SARSCoV2-030 | MT787748 | hCoV-19/Turkey/KU-030/2020 | EPI_ISL_495440 | B.1 (G) |
| Kafkas-SARSCoV2-031 | MT787742 | hCoV-19/Turkey/KU-031/2020 | EPI_ISL_495441 | B.1.1 (GR) |
| Kafkas-SARSCoV2-032 | MT787486 | hCoV-19/Turkey/KU-032/2020 | EPI_ISL_495442 | B.1.9 (GH) |
| Kafkas-SARSCoV2-033 | MT787553 | hCoV-19/Turkey/KU-033/2020 | EPI_ISL_495443 | B.1.9 (GH) |
| Kafkas-SARSCoV2-034 | MT787473 | hCoV-19/Turkey/KU-034/2020 | EPI_ISL_495444 | B.1.1 (GR) |
| Kafkas-SARSCoV2-035 | MT787747 | hCoV-19/Turkey/KU-035/2020 | EPI_ISL_495445 | B.1.9 (GH) |
| Kafkas-SARSCoV2-036 | MT787743 | hCoV-19/Turkey/KU-036/2020 | EPI_ISL_495446 | B.1.1 (GR) |
| Kafkas-SARSCoV2-037 | MT787506 | hCoV-19/Turkey/KU-037/2020 | EPI_ISL_495447 | B.1.1 (GR) |
| Kafkas-SARSCoV2-038 | MT789691 | hCoV-19/Turkey/KU-038/2020 | EPI_ISL_495448 | B.1.9 (GH) |
| Kafkas-SARSCoV2-039 | MT787744 | hCoV-19/Turkey/KU-039/2020 | EPI_ISL_495449 | B.1.1 (GR) |
| Kafkas-SARSCoV2-040 | MT787746 | hCoV-19/Turkey/KU-040/2020 | EPI_ISL_495450 | B.1.1 (GR) |
| Kafkas-SARSCoV2-041 | MT789690 | hCoV-19/Turkey/KU-041/2020 | EPI_ISL_495451 | B.1 (GH) |
| Kafkas-SARSCoV2-042 | MT787503 | hCoV-19/Turkey/KU-042/2020 | EPI_ISL_495452 | B.1.1 (GR) |
| Kafkas-SARSCoV2-043 | MT789688 | hCoV-19/Turkey/KU-043/2020 | EPI_ISL_495453 | B.1.1 (GR) |
| Kafkas-SARSCoV2-044 | MT789692 | hCoV-19/Turkey/KU-044/2020 | EPI_ISL_495454 | B.1.1 (GR) |
| Kafkas-SARSCoV2-045 | MT787558 | hCoV-19/Turkey/KU-045/2020 | EPI_ISL_495455 | B.1.9 (GH) |
| Kafkas-SARSCoV2-046 | MT789694 | hCoV-19/Turkey/KU-046/2020 | EPI_ISL_495456 | B.1.1 (GR) |
| Kafkas-SARSCoV2-047 | MT787487 | hCoV-19/Turkey/KU-047/2020 | EPI_ISL_495457 | B.1.9 (GH) |
