# Supplementary material for: Molecular characterization, phylogenetic and variation analyses of SARS-CoV-2 strains in Turkey
Source: Future Microbiol. 2021 Oct 7:10.2217/fmb-2021-0118. doi: 10.2217/fmb-2021-0118 (PMC8507978; doi:10.2217/fmb-2021-0118)
Supplement: Supplementary file 2 [file Supplementary_Table_2.docx]

**Supplementary Table 2:** Coding and non-coding mutation list detected in SARS-CoV-2 genomes of our isolates.

| **Sample ID** | **GenBank Accession** | **GISAID**  **Accession** | **Genome position** | **Gene** | **Final product** | **Protein** | **Nucleotide variation** | **Mutation type** | **n** |
| --- | --- | --- | --- | --- | --- | --- | --- | --- | --- |
| Kafkas-SARSCoV2-001 | MT786327 | EPI_ISL_495411 | 241 | 5'UTR | NA | NA | c.1-25C>T | Non-coding | 47 |
| Kafkas-SARSCoV2-001 |  |  | 313 | ORF1ab | leader protein | p.(Leu16=) | c.48C>T | synonymous | 22 |
| Kafkas-SARSCoV2-001 |  |  | 2509 | ORF1ab | nsp2 | p.(Pro748=) | c.2244C>T | synonymous | 22 |
| Kafkas-SARSCoV2-001 |  |  | 3037 | ORF1ab | nsp3 | p.(Phe924=) | c.2772C>T | synonymous | 47 |
| Kafkas-SARSCoV2-001 |  |  | 13620 | ORF1ab | RNA-dep RNA-pol | p.(Asp4452=) | c.13355C>T | synonymous | 21 |
| Kafkas-SARSCoV2-001 |  |  | 14408 | ORF1ab | RNA-dep RNA-pol | p.(Pro4715Leu) | c.14143C>T | missense | 47 |
| Kafkas-SARSCoV2-001 |  |  | 14724 | ORF1ab | RNA-dep RNA-pol | p.(Phe4820=) | c.14459C>T | synonymous | 22 |
| Kafkas-SARSCoV2-001 |  |  | 23403 | S | NA | p.(Asp614Gly) | c.1841A>G | missense | 47 |
| Kafkas-SARSCoV2-001 |  |  | 24262 | S | NA | p.(Met900Ile) | c.2700G>T | missense | 23 |
| Kafkas-SARSCoV2-001 |  |  | 27703 | ORF7a | NA | p.(Val104Phe) | c.310G>T | missense | 23 |
| Kafkas-SARSCoV2-001 |  |  | 28881 | N | NA | p.(Arg203Lys) | c.608G>A | missense | 28 |
| Kafkas-SARSCoV2-001 |  |  | 28882 | N | NA | p.(Arg203=) | c.609G>A | synonymous | 28 |
| Kafkas-SARSCoV2-001 |  |  | 28883 | N | NA | p.(Gly204Arg) | c.610G>C | missense | 28 |
| Kafkas-SARSCoV2-002 | MT786861 | EPI_ISL_495412 | 241 | 5'UTR | NA | NA | c.1-25C>T | Non-coding | 47 |
| Kafkas-SARSCoV2-002 |  |  | 313 | ORF1ab | leader protein | p.(Leu16=) | c.48C>T | synonymous | 22 |
| Kafkas-SARSCoV2-002 |  |  | 2509 | ORF1ab | nsp2 | p.(Pro748=) | c.2244C>T | synonymous | 22 |
| Kafkas-SARSCoV2-002 |  |  | 3037 | ORF1ab | nsp3 | p.(Phe924=) | c.2772C>T | synonymous | 47 |
| Kafkas-SARSCoV2-002 |  |  | 5015 | ORF1ab | nsp3 | p.(Val1584Met) | c.4750G>A | missense | 13 |
| Kafkas-SARSCoV2-002 |  |  | 13620 | ORF1ab | RNA-dep RNA-pol | p.(Asp4452=) | c.13355C>T | synonymous | 21 |
| Kafkas-SARSCoV2-002 |  |  | 14408 | ORF1ab | RNA-dep RNA-pol | p.(Pro4715Leu) | c.14143C>T | missense | 47 |
| Kafkas-SARSCoV2-002 |  |  | 14724 | ORF1ab | RNA-dep RNA-pol | p.(Phe4820=) | c.14459C>T | synonymous | 22 |
| Kafkas-SARSCoV2-002 |  |  | 23403 | S | NA | p.(Asp614Gly) | c.1841A>G | missense | 47 |
| Kafkas-SARSCoV2-002 |  |  | 24262 | S | NA | p.(Met900Ile) | c.2700G>T | missense | 23 |
| Kafkas-SARSCoV2-002 |  |  | 27703 | ORF7a | NA | p.(Val104Phe) | c.310G>T | missense | 23 |
| Kafkas-SARSCoV2-002 |  |  | 28881 | N | NA | p.(Arg203Lys) | c.608G>A | missense | 28 |
| Kafkas-SARSCoV2-002 |  |  | 28882 | N | NA | p.(Arg203=) | c.609G>A | synonymous | 28 |
| Kafkas-SARSCoV2-002 |  |  | 28883 | N | NA | p.(Gly204Arg) | c.610G>C | missense | 28 |
| Kafkas-SARSCoV2-003 | MT786865 | EPI_ISL_495413 | 241 | 5'UTR | NA | NA | c.1-25C>T | Non-coding | 47 |
| Kafkas-SARSCoV2-003 |  |  | 3037 | ORF1ab | nsp3 | p.(Phe924=) | c.2772C>T | synonymous | 47 |
| Kafkas-SARSCoV2-003 |  |  | 14408 | ORF1ab | RNA-dep RNA-pol | p.(Pro4715Leu) | c.14143C>T | missense | 47 |
| Kafkas-SARSCoV2-003 |  |  | 23403 | S | NA | p.(Asp614Gly) | c.1841A>G | missense | 47 |
| Kafkas-SARSCoV2-003 |  |  | 24262 | S | NA | p.(Met900Ile) | c.2700G>T | missense | 23 |
| Kafkas-SARSCoV2-003 |  |  | 27703 | ORF7a | NA | p.(Val104Phe) | c.310G>T | missense | 23 |
| Kafkas-SARSCoV2-003 |  |  | 28881 | N | NA | p.(Arg203Lys) | c.608G>A | missense | 28 |
| Kafkas-SARSCoV2-003 |  |  | 28882 | N | NA | p.(Arg203=) | c.609G>A | synonymous | 28 |
| Kafkas-SARSCoV2-003 |  |  | 28883 | N | NA | p.(Gly204Arg) | c.610G>C | missense | 28 |
| Kafkas-SARSCoV2-004 | MT786329 | EPI_ISL_495414 | 241 | 5'UTR | NA | NA | c.1-25C>T | Non-coding | 47 |
| Kafkas-SARSCoV2-004 |  |  | 3037 | ORF1ab | nsp3 | p.(Phe924=) | c.2772C>T | synonymous | 47 |
| Kafkas-SARSCoV2-004 |  |  | 7765 | ORF1ab | nsp3 | p.(Ser2500=) | c.7500C>T | synonymous | 14 |
| Kafkas-SARSCoV2-004 |  |  | 8326 | ORF1ab | nsp3 | p.(Asp2687=) | c.8061C>T | synonymous | 14 |
| Kafkas-SARSCoV2-004 |  |  | 14408 | ORF1ab | RNA-dep RNA-pol | p.(Pro4715Leu) | c.14143C>T | missense | 47 |
| Kafkas-SARSCoV2-004 |  |  | 17690 | ORF1ab | helicase | p.(Ser5809Leu) | c.17425C>T | missense | 14 |
| Kafkas-SARSCoV2-004 |  |  | 18877 | ORF1ab | 3'-to-5' exonuclease | p.(Leu6205=) | c.18612C>T | synonymous | 15 |
| Kafkas-SARSCoV2-004 |  |  | 23403 | S | NA | p.(Asp614Gly) | c.1841A>G | missense | 47 |
| Kafkas-SARSCoV2-004 |  |  | 25563 | ORF3a | NA | p.(Gln57His) | c.171G>T | missense | 16 |
| Kafkas-SARSCoV2-005 | MT786797 | EPI_ISL_495415 | 241 | 5'UTR | NA | NA | c.1-25C>T | Non-coding | 47 |
| Kafkas-SARSCoV2-005 |  |  | 313 | ORF1ab | leader protein | p.(Leu16=) | c.48C>T | synonymous | 22 |
| Kafkas-SARSCoV2-005 |  |  | 2509 | ORF1ab | nsp2 | p.(Pro748=) | c.2244C>T | synonymous | 22 |
| Kafkas-SARSCoV2-005 |  |  | 3037 | ORF1ab | nsp3 | p.(Phe924=) | c.2772C>T | synonymous | 47 |
| Kafkas-SARSCoV2-005 |  |  | 5015 | ORF1ab | nsp3 | p.(Val1584Met) | c.4750G>A | missense | 13 |
| Kafkas-SARSCoV2-005 |  |  | 13620 | ORF1ab | RNA-dep RNA-pol | p.(Asp4452=) | c.13355C>T | synonymous | 21 |
| Kafkas-SARSCoV2-005 |  |  | 14408 | ORF1ab | RNA-dep RNA-pol | p.(Pro4715Leu) | c.14143C>T | missense | 47 |
| Kafkas-SARSCoV2-005 |  |  | 14724 | ORF1ab | RNA-dep RNA-pol | p.(Phe4820=) | c.14459C>T | synonymous | 22 |
| Kafkas-SARSCoV2-005 |  |  | 23403 | S | NA | p.(Asp614Gly) | c.1841A>G | missense | 47 |
| Kafkas-SARSCoV2-005 |  |  | 24262 | S | NA | p.(Met900Ile) | c.2700G>T | missense | 23 |
| Kafkas-SARSCoV2-005 |  |  | 27703 | ORF7a | NA | p.(Val104Phe) | c.310G>T | missense | 23 |
| Kafkas-SARSCoV2-005 |  |  | 28881 | N | NA | p.(Arg203Lys) | c.608G>A | missense | 28 |
| Kafkas-SARSCoV2-005 |  |  | 28882 | N | NA | p.(Arg203=) | c.609G>A | synonymous | 28 |
| Kafkas-SARSCoV2-005 |  |  | 28883 | N | NA | p.(Gly204Arg) | c.610G>C | missense | 28 |
| Kafkas-SARSCoV2-006 | MT786331 | EPI_ISL_495416 | 241 | 5'UTR | NA | NA | c.1-25C>T | Non-coding | 47 |
| Kafkas-SARSCoV2-006 |  |  | 313 | ORF1ab | leader protein | p.(Leu16=) | c.48C>T | synonymous | 22 |
| Kafkas-SARSCoV2-006 |  |  | 2509 | ORF1ab | nsp2 | p.(Pro748=) | c.2244C>T | synonymous | 22 |
| Kafkas-SARSCoV2-006 |  |  | 3037 | ORF1ab | nsp3 | p.(Phe924=) | c.2772C>T | synonymous | 47 |
| Kafkas-SARSCoV2-006 |  |  | 13620 | ORF1ab | RNA-dep RNA-pol | p.(Asp4452=) | c.13355C>T | synonymous | 21 |
| Kafkas-SARSCoV2-006 |  |  | 14408 | ORF1ab | RNA-dep RNA-pol | p.(Pro4715Leu) | c.14143C>T | missense | 47 |
| Kafkas-SARSCoV2-006 |  |  | 14724 | ORF1ab | RNA-dep RNA-pol | p.(Phe4820=) | c.14459C>T | synonymous | 22 |
| Kafkas-SARSCoV2-006 |  |  | 23403 | S | NA | p.(Asp614Gly) | c.1841A>G | missense | 47 |
| Kafkas-SARSCoV2-006 |  |  | 24262 | S | NA | p.(Met900Ile) | c.2700G>T | missense | 23 |
| Kafkas-SARSCoV2-006 |  |  | 27703 | ORF7a | NA | p.(Val104Phe) | c.310G>T | missense | 23 |
| Kafkas-SARSCoV2-006 |  |  | 28881 | N | NA | p.(Arg203Lys) | c.608G>A | missense | 28 |
| Kafkas-SARSCoV2-006 |  |  | 28882 | N | NA | p.(Arg203=) | c.609G>A | synonymous | 28 |
| Kafkas-SARSCoV2-006 |  |  | 28883 | N | NA | p.(Gly204Arg) | c.610G>C | missense | 28 |
| Kafkas-SARSCoV2-007 | MT786334 | EPI_ISL_495417 | 241 | 5'UTR | NA | NA | c.1-25C>T | Non-coding | 47 |
| Kafkas-SARSCoV2-007 |  |  | 3037 | ORF1ab | nsp3 | p.(Phe924=) | c.2772C>T | synonymous | 47 |
| Kafkas-SARSCoV2-007 |  |  | 6317 | ORF1ab | nsp3 | p.(Pro2018Ser) | c.6052C>T | missense | 1 |
| Kafkas-SARSCoV2-007 |  |  | 7765 | ORF1ab | nsp3 | p.(Ser2500=) | c.7500C>T | synonymous | 14 |
| Kafkas-SARSCoV2-007 |  |  | 8326 | ORF1ab | nsp3 | p.(Asp2687=) | c.8061C>T | synonymous | 14 |
| Kafkas-SARSCoV2-007 |  |  | 14408 | ORF1ab | RNA-dep RNA-pol | p.(Pro4715Leu) | c.14143C>T | missense | 47 |
| Kafkas-SARSCoV2-007 |  |  | 17690 | ORF1ab | helicase | p.(Ser5809Leu) | c.17425C>T | missense | 14 |
| Kafkas-SARSCoV2-007 |  |  | 18877 | ORF1ab | 3'-to-5' exonuclease | p.(Leu6205=) | c.18612C>T | synonymous | 15 |
| Kafkas-SARSCoV2-007 |  |  | 23403 | S | NA | p.(Asp614Gly) | c.1841A>G | missense | 47 |
| Kafkas-SARSCoV2-007 |  |  | 25563 | ORF3a | NA | p.(Gln57His) | c.171G>T | missense | 16 |
| Kafkas-SARSCoV2-008 | MT786859 | EPI_ISL_495418 | 241 | 5'UTR | NA | NA | c.1-25C>T | Non-coding | 47 |
| Kafkas-SARSCoV2-008 |  |  | 313 | ORF1ab | leader protein | p.(Leu16=) | c.48C>T | synonymous | 22 |
| Kafkas-SARSCoV2-008 |  |  | 2509 | ORF1ab | nsp2 | p.(Pro748=) | c.2244C>T | synonymous | 22 |
| Kafkas-SARSCoV2-008 |  |  | 3037 | ORF1ab | nsp3 | p.(Phe924=) | c.2772C>T | synonymous | 47 |
| Kafkas-SARSCoV2-008 |  |  | 13620 | ORF1ab | RNA-dep RNA-pol | p.(Asp4452=) | c.13355C>T | synonymous | 21 |
| Kafkas-SARSCoV2-008 |  |  | 14408 | ORF1ab | RNA-dep RNA-pol | p.(Pro4715Leu) | c.14143C>T | missense | 47 |
| Kafkas-SARSCoV2-008 |  |  | 14724 | ORF1ab | RNA-dep RNA-pol | p.(Phe4820=) | c.14459C>T | synonymous | 22 |
| Kafkas-SARSCoV2-008 |  |  | 23403 | S | NA | p.(Asp614Gly) | c.1841A>G | missense | 47 |
| Kafkas-SARSCoV2-008 |  |  | 24262 | S | NA | p.(Met900Ile) | c.2700G>T | missense | 23 |
| Kafkas-SARSCoV2-008 |  |  | 27703 | ORF7a | NA | p.(Val104Phe) | c.310G>T | missense | 23 |
| Kafkas-SARSCoV2-008 |  |  | 28881 | N | NA | p.(Arg203Lys) | c.608G>A | missense | 28 |
| Kafkas-SARSCoV2-008 |  |  | 28882 | N | NA | p.(Arg203=) | c.609G>A | synonymous | 28 |
| Kafkas-SARSCoV2-008 |  |  | 28883 | N | NA | p.(Gly204Arg) | c.610G>C | missense | 28 |
| Kafkas-SARSCoV2-009 | MT786860 | EPI_ISL_495419 | 241 | 5'UTR | NA | NA | c.1-25C>T | Non-coding | 47 |
| Kafkas-SARSCoV2-009 |  |  | 3037 | ORF1ab | nsp3 | p.(Phe924=) | c.2772C>T | synonymous | 47 |
| Kafkas-SARSCoV2-009 |  |  | 14408 | ORF1ab | RNA-dep RNA-pol | p.(Pro4715Leu) | c.14143C>T | missense | 47 |
| Kafkas-SARSCoV2-009 |  |  | 23403 | S | NA | p.(Asp614Gly) | c.1841A>G | missense | 47 |
| Kafkas-SARSCoV2-010 | MT786335 | EPI_ISL_49542 | 241 | 5'UTR | NA | NA | c.1-25C>T | Non-coding | 47 |
| Kafkas-SARSCoV2-010 |  |  | 313 | ORF1ab | leader protein | p.(Leu16=) | c.48C>T | synonymous | 22 |
| Kafkas-SARSCoV2-010 |  |  | 2509 | ORF1ab | nsp2 | p.(Pro748=) | c.2244C>T | synonymous | 22 |
| Kafkas-SARSCoV2-010 |  |  | 3037 | ORF1ab | nsp3 | p.(Phe924=) | c.2772C>T | synonymous | 47 |
| Kafkas-SARSCoV2-010 |  |  | 4465 | ORF1ab | nsp3 | p.(Ser1400=) | c.4200A>G | synonymous | 1 |
| Kafkas-SARSCoV2-010 |  |  | 13620 | ORF1ab | RNA-dep RNA-pol | p.(Asp4452=) | c.13355C>T | synonymous | 21 |
| Kafkas-SARSCoV2-010 |  |  | 14408 | ORF1ab | RNA-dep RNA-pol | p.(Pro4715Leu) | c.14143C>T | missense | 47 |
| Kafkas-SARSCoV2-010 |  |  | 14724 | ORF1ab | RNA-dep RNA-pol | p.(Phe4820=) | c.14459C>T | synonymous | 22 |
| Kafkas-SARSCoV2-010 |  |  | 23403 | S | NA | p.(Asp614Gly) | c.1841A>G | missense | 47 |
| Kafkas-SARSCoV2-010 |  |  | 24262 | S | NA | p.(Met900Ile) | c.2700G>T | missense | 23 |
| Kafkas-SARSCoV2-010 |  |  | 27703 | ORF7a | NA | p.(Val104Phe) | c.310G>T | missense | 23 |
| Kafkas-SARSCoV2-010 |  |  | 28881 | N | NA | p.(Arg203Lys) | c.608G>A | missense | 28 |
| Kafkas-SARSCoV2-010 |  |  | 28882 | N | NA | p.(Arg203=) | c.609G>A | synonymous | 28 |
| Kafkas-SARSCoV2-010 |  |  | 28883 | N | NA | p.(Gly204Arg) | c.610G>C | missense | 28 |
| Kafkas-SARSCoV2-011 | MT786868 | EPI_ISL_495421 | 241 | 5'UTR | NA | NA | c.1-25C>T | Non-coding | 47 |
| Kafkas-SARSCoV2-011 |  |  | 313 | ORF1ab | leader protein | p.(Leu16=) | c.48C>T | synonymous | 22 |
| Kafkas-SARSCoV2-011 |  |  | 2509 | ORF1ab | nsp2 | p.(Pro748=) | c.2244C>T | synonymous | 22 |
| Kafkas-SARSCoV2-011 |  |  | 3037 | ORF1ab | nsp3 | p.(Phe924=) | c.2772C>T | synonymous | 47 |
| Kafkas-SARSCoV2-011 |  |  | 4148 | ORF1ab | nsp3 | p.(Val1295Phe) | c.3883G>T | missense | 2 |
| Kafkas-SARSCoV2-011 |  |  | 5015 | ORF1ab | nsp3 | p.(Val1584Met) | c.4750G>A | missense | 13 |
| Kafkas-SARSCoV2-011 |  |  | 13620 | ORF1ab | RNA-dep RNA-pol | p.(Asp4452=) | c.13355C>T | synonymous | 21 |
| Kafkas-SARSCoV2-011 |  |  | 14408 | ORF1ab | RNA-dep RNA-pol | p.(Pro4715Leu) | c.14143C>T | missense | 47 |
| Kafkas-SARSCoV2-011 |  |  | 14724 | ORF1ab | RNA-dep RNA-pol | p.(Phe4820=) | c.14459C>T | synonymous | 22 |
| Kafkas-SARSCoV2-011 |  |  | 23403 | S | NA | p.(Asp614Gly) | c.1841A>G | missense | 47 |
| Kafkas-SARSCoV2-011 |  |  | 24262 | S | NA | p.(Met900Ile) | c.2700G>T | missense | 23 |
| Kafkas-SARSCoV2-011 |  |  | 27703 | ORF7a | NA | p.(Val104Phe) | c.310G>T | missense | 23 |
| Kafkas-SARSCoV2-011 |  |  | 28166 | ORF8 | NA | p.(Gln91=) | c.273G>A | synonymous | 2 |
| Kafkas-SARSCoV2-011 |  |  | 28881 | N | NA | p.(Arg203Lys) | c.608G>A | missense | 28 |
| Kafkas-SARSCoV2-011 |  |  | 28882 | N | NA | p.(Arg203=) | c.609G>A | synonymous | 28 |
| Kafkas-SARSCoV2-011 |  |  | 28883 | N | NA | p.(Gly204Arg) | c.610G>C | missense | 28 |
| Kafkas-SARSCoV2-012 | MT787647 | EPI_ISL_495422 | 241 | 5'UTR | NA | NA | c.1-25C>T | Non-coding | 47 |
| Kafkas-SARSCoV2-012 |  |  | 313 | ORF1ab | leader protein | p.(Leu16=) | c.48C>T | synonymous | 22 |
| Kafkas-SARSCoV2-012 |  |  | 2509 | ORF1ab | nsp2 | p.(Pro748=) | c.2244C>T | synonymous | 22 |
| Kafkas-SARSCoV2-012 |  |  | 3037 | ORF1ab | nsp3 | p.(Phe924=) | c.2772C>T | synonymous | 47 |
| Kafkas-SARSCoV2-012 |  |  | 13620 | ORF1ab | RNA-dep RNA-pol | p.(Asp4452=) | c.13355C>T | synonymous | 21 |
| Kafkas-SARSCoV2-012 |  |  | 14408 | ORF1ab | RNA-dep RNA-pol | p.(Pro4715Leu) | c.14143C>T | missense | 47 |
| Kafkas-SARSCoV2-012 |  |  | 14724 | ORF1ab | RNA-dep RNA-pol | p.(Phe4820=) | c.14459C>T | synonymous | 22 |
| Kafkas-SARSCoV2-012 |  |  | 23403 | S | NA | p.(Asp614Gly) | c.1841A>G | missense | 47 |
| Kafkas-SARSCoV2-012 |  |  | 24262 | S | NA | p.(Met900Ile) | c.2700G>T | missense | 23 |
| Kafkas-SARSCoV2-012 |  |  | 27703 | ORF7a | NA | p.(Val104Phe) | c.310G>T | missense | 23 |
| Kafkas-SARSCoV2-012 |  |  | 28881 | N | NA | p.(Arg203Lys) | c.608G>A | missense | 28 |
| Kafkas-SARSCoV2-012 |  |  | 28882 | N | NA | p.(Arg203=) | c.609G>A | synonymous | 28 |
| Kafkas-SARSCoV2-012 |  |  | 28883 | N | NA | p.(Gly204Arg) | c.610G>C | missense | 28 |
| Kafkas-SARSCoV2-013 | MT787564 | EPI_ISL_495423 | 241 | 5'UTR | NA | NA | c.1-25C>T | Non-coding | 47 |
| Kafkas-SARSCoV2-013 |  |  | 3037 | ORF1ab | nsp3 | p.(Phe924=) | c.2772C>T | synonymous | 47 |
| Kafkas-SARSCoV2-013 |  |  | 4795 | ORF1ab | nsp3 | p.(Ser1510=) | c.4530C>T | synonymous | 1 |
| Kafkas-SARSCoV2-013 |  |  | 11083 | ORF1ab | nsp6 | p.(Leu3606Phe) | c.10818G>T | missense | 3 |
| Kafkas-SARSCoV2-013 |  |  | 12809 | ORF1ab | nsp9 | p.(Leu4182Phe) | c.12544C>T | missense | 3 |
| Kafkas-SARSCoV2-013 |  |  | 14408 | ORF1ab | RNA-dep RNA-pol | p.(Pro4715Leu) | c.14143C>T | missense | 47 |
| Kafkas-SARSCoV2-013 |  |  | 23403 | S | NA | p.(Asp614Gly) | c.1841A>G | missense | 47 |
| Kafkas-SARSCoV2-013 |  |  | 28881 | N | NA | p.(Arg203Lys) | c.608G>A | missense | 28 |
| Kafkas-SARSCoV2-013 |  |  | 28882 | N | NA | p.(Arg203=) | c.609G>A | synonymous | 28 |
| Kafkas-SARSCoV2-013 |  |  | 28883 | N | NA | p.(Gly204Arg) | c.610G>C | missense | 28 |
| Kafkas-SARSCoV2-013 |  |  | 29171 | N | NA | p.(His300Tyr) | c.898C>T | missense | 2 |
| Kafkas-SARSCoV2-014 | MT786866 | EPI_ISL_495424 | 241 | 5'UTR | NA | NA | c.1-25C>T | Non-coding | 47 |
| Kafkas-SARSCoV2-014 |  |  | 313 | ORF1ab | leader protein | p.(Leu16=) | c.48C>T | synonymous | 22 |
| Kafkas-SARSCoV2-014 |  |  | 2509 | ORF1ab | nsp2 | p.(Pro748=) | c.2244C>T | synonymous | 22 |
| Kafkas-SARSCoV2-014 |  |  | 3037 | ORF1ab | nsp3 | p.(Phe924=) | c.2772C>T | synonymous | 47 |
| Kafkas-SARSCoV2-014 |  |  | 13620 | ORF1ab | RNA-dep RNA-pol | p.(Asp4452=) | c.13355C>T | synonymous | 21 |
| Kafkas-SARSCoV2-014 |  |  | 14408 | ORF1ab | RNA-dep RNA-pol | p.(Pro4715Leu) | c.14143C>T | missense | 47 |
| Kafkas-SARSCoV2-014 |  |  | 14724 | ORF1ab | RNA-dep RNA-pol | p.(Phe4820=) | c.14459C>T | synonymous | 22 |
| Kafkas-SARSCoV2-014 |  |  | 23403 | S | NA | p.(Asp614Gly) | c.1841A>G | missense | 47 |
| Kafkas-SARSCoV2-014 |  |  | 24262 | S | NA | p.(Met900Ile) | c.2700G>T | missense | 23 |
| Kafkas-SARSCoV2-014 |  |  | 27703 | ORF7a | NA | p.(Val104Phe) | c.310G>T | missense | 23 |
| Kafkas-SARSCoV2-014 |  |  | 28881 | N | NA | p.(Arg203Lys) | c.608G>A | missense | 28 |
| Kafkas-SARSCoV2-014 |  |  | 28882 | N | NA | p.(Arg203=) | c.609G>A | synonymous | 28 |
| Kafkas-SARSCoV2-014 |  |  | 28883 | N | NA | p.(Gly204Arg) | c.610G>C | missense | 28 |
| Kafkas-SARSCoV2-015 | MT787648 | EPI_ISL_495425 | 241 | 5'UTR | NA | NA | c.1-25C>T | Non-coding | 47 |
| Kafkas-SARSCoV2-015 |  |  | 313 | ORF1ab | leader protein | p.(Leu16=) | c.48C>T | synonymous | 22 |
| Kafkas-SARSCoV2-015 |  |  | 2509 | ORF1ab | nsp2 | p.(Pro748=) | c.2244C>T | synonymous | 22 |
| Kafkas-SARSCoV2-015 |  |  | 3037 | ORF1ab | nsp3 | p.(Phe924=) | c.2772C>T | synonymous | 47 |
| Kafkas-SARSCoV2-015 |  |  | 5015 | ORF1ab | nsp3 | p.(Val1584Met) | c.4750G>A | missense | 13 |
| Kafkas-SARSCoV2-015 |  |  | 13620 | ORF1ab | RNA-dep RNA-pol | p.(Asp4452=) | c.13355C>T | synonymous | 21 |
| Kafkas-SARSCoV2-015 |  |  | 14408 | ORF1ab | RNA-dep RNA-pol | p.(Pro4715Leu) | c.14143C>T | missense | 47 |
| Kafkas-SARSCoV2-015 |  |  | 14724 | ORF1ab | RNA-dep RNA-pol | p.(Phe4820=) | c.14459C>T | synonymous | 22 |
| Kafkas-SARSCoV2-015 |  |  | 23403 | S | NA | p.(Asp614Gly) | c.1841A>G | missense | 47 |
| Kafkas-SARSCoV2-015 |  |  | 24262 | S | NA | p.(Met900Ile) | c.2700G>T | missense | 23 |
| Kafkas-SARSCoV2-015 |  |  | 27703 | ORF7a | NA | p.(Val104Phe) | c.310G>T | missense | 23 |
| Kafkas-SARSCoV2-015 |  |  | 28881 | N | NA | p.(Arg203Lys) | c.608G>A | missense | 28 |
| Kafkas-SARSCoV2-015 |  |  | 28882 | N | NA | p.(Arg203=) | c.609G>A | synonymous | 28 |
| Kafkas-SARSCoV2-015 |  |  | 28883 | N | NA | p.(Gly204Arg) | c.610G>C | missense | 28 |
| Kafkas-SARSCoV2-016 | MT787650 | EPI_ISL_495426 | 241 | 5'UTR | NA | NA | c.1-25C>T | Non-coding | 47 |
| Kafkas-SARSCoV2-016 |  |  | 1059 | ORF1ab | nsp2 | p.(Thr265Ile) | c.794C>T | missense | 1 |
| Kafkas-SARSCoV2-016 |  |  | 3037 | ORF1ab | nsp3 | p.(Phe924=) | c.2772C>T | synonymous | 47 |
| Kafkas-SARSCoV2-016 |  |  | 4886 | ORF1ab | nsp3 | p.(Pro1541Ser) | c.4621C>T | missense | 1 |
| Kafkas-SARSCoV2-016 |  |  | 11083 | ORF1ab | nsp6 | p.(Leu3606Phe) | c.10818G>T | missense | 3 |
| Kafkas-SARSCoV2-016 |  |  | 13326 | ORF1ab | nsp10 | p.(Thr4354Ile) | c.13061C>T | missense | 1 |
| Kafkas-SARSCoV2-016 |  |  | 14408 | ORF1ab | RNA-dep RNA-pol | p.(Pro4715Leu) | c.14143C>T | missense | 47 |
| Kafkas-SARSCoV2-016 |  |  | 14937 | ORF1ab | RNA-dep RNA-pol | p.(Asp4891=) | c.14672C>T | synonymous | 1 |
| Kafkas-SARSCoV2-016 |  |  | 16914 | ORF1ab | helicase | p.(Val5550=) | c.16649G>T | synonymous | 1 |
| Kafkas-SARSCoV2-016 |  |  | 23403 | S | NA | p.(Asp614Gly) | c.1841A>G | missense | 47 |
| Kafkas-SARSCoV2-016 |  |  | 25563 | ORF3a | NA | p.(Gln57His) | c.171G>T | missense | 16 |
| Kafkas-SARSCoV2-016 |  |  | 26814 | M | NA | p.(Ala98Ser) | c.292G>T | missense | 1 |
| Kafkas-SARSCoV2-017 | MT787578 | EPI_ISL_495427 | 241 | 5'UTR | NA | NA | c.1-25C>T | Non-coding | 47 |
| Kafkas-SARSCoV2-017 |  |  | 3037 | ORF1ab | nsp3 | p.(Phe924=) | c.2772C>T | synonymous | 47 |
| Kafkas-SARSCoV2-017 |  |  | 14408 | ORF1ab | RNA-dep RNA-pol | p.(Pro4715Leu) | c.14143C>T | missense | 47 |
| Kafkas-SARSCoV2-017 |  |  | 23403 | S | NA | p.(Asp614Gly) | c.1841A>G | missense | 47 |
| Kafkas-SARSCoV2-018 | MT787645 | EPI_ISL_495428 | 241 | 5'UTR | NA | NA | c.1-25C>T | Non-coding | 47 |
| Kafkas-SARSCoV2-018 |  |  | 3037 | ORF1ab | nsp3 | p.(Phe924=) | c.2772C>T | synonymous | 47 |
| Kafkas-SARSCoV2-018 |  |  | 7765 | ORF1ab | nsp3 | p.(Ser2500=) | c.7500C>T | synonymous | 14 |
| Kafkas-SARSCoV2-018 |  |  | 8326 | ORF1ab | nsp3 | p.(Asp2687=) | c.8061C>T | synonymous | 14 |
| Kafkas-SARSCoV2-018 |  |  | 14408 | ORF1ab | RNA-dep RNA-pol | p.(Pro4715Leu) | c.14143C>T | missense | 47 |
| Kafkas-SARSCoV2-018 |  |  | 17690 | ORF1ab | helicase | p.(Ser5809Leu) | c.17425C>T | missense | 14 |
| Kafkas-SARSCoV2-018 |  |  | 18877 | ORF1ab | 3'-to-5' exonuclease | p.(Leu6205=) | c.18612C>T | synonymous | 15 |
| Kafkas-SARSCoV2-018 |  |  | 23403 | S | NA | p.(Asp614Gly) | c.1841A>G | missense | 47 |
| Kafkas-SARSCoV2-018 |  |  | 25563 | ORF3a | NA | p.(Gln57His) | c.171G>T | missense | 16 |
| Kafkas-SARSCoV2-019 | MT787577 | EPI_ISL_495429 | 241 | 5'UTR | NA | NA | c.1-25C>T | Non-coding | 47 |
| Kafkas-SARSCoV2-019 |  |  | 3037 | ORF1ab | nsp3 | p.(Phe924=) | c.2772C>T | synonymous | 47 |
| Kafkas-SARSCoV2-019 |  |  | 7765 | ORF1ab | nsp3 | p.(Ser2500=) | c.7500C>T | synonymous | 14 |
| Kafkas-SARSCoV2-019 |  |  | 8326 | ORF1ab | nsp3 | p.(Asp2687=) | c.8061C>T | synonymous | 14 |
| Kafkas-SARSCoV2-019 |  |  | 14408 | ORF1ab | RNA-dep RNA-pol | p.(Pro4715Leu) | c.14143C>T | missense | 47 |
| Kafkas-SARSCoV2-019 |  |  | 17690 | ORF1ab | helicase | p.(Ser5809Leu) | c.17425C>T | missense | 14 |
| Kafkas-SARSCoV2-019 |  |  | 18877 | ORF1ab | 3'-to-5' exonuclease | p.(Leu6205=) | c.18612C>T | synonymous | 15 |
| Kafkas-SARSCoV2-019 |  |  | 23403 | S | NA | p.(Asp614Gly) | c.1841A>G | missense | 47 |
| Kafkas-SARSCoV2-019 |  |  | 25563 | ORF3a | NA | p.(Gln57His) | c.171G>T | missense | 16 |
| Kafkas-SARSCoV2-020 | MT787646 | EPI_ISL_495430 | 241 | 5'UTR | NA | NA | c.1-25C>T | Non-coding | 47 |
| Kafkas-SARSCoV2-020 |  |  | 3037 | ORF1ab | nsp3 | p.(Phe924=) | c.2772C>T | synonymous | 47 |
| Kafkas-SARSCoV2-020 |  |  | 7765 | ORF1ab | nsp3 | p.(Ser2500=) | c.7500C>T | synonymous | 14 |
| Kafkas-SARSCoV2-020 |  |  | 8326 | ORF1ab | nsp3 | p.(Asp2687=) | c.8061C>T | synonymous | 14 |
| Kafkas-SARSCoV2-020 |  |  | 14408 | ORF1ab | RNA-dep RNA-pol | p.(Pro4715Leu) | c.14143C>T | missense | 47 |
| Kafkas-SARSCoV2-020 |  |  | 16428 | ORF1ab | helicase | p.(Tyr5388=) | c.16163C>T | synonymous | 6 |
| Kafkas-SARSCoV2-020 |  |  | 17690 | ORF1ab | helicase | p.(Ser5809Leu) | c.17425C>T | missense | 14 |
| Kafkas-SARSCoV2-020 |  |  | 18877 | ORF1ab | 3'-to-5' exonuclease | p.(Leu6205=) | c.18612C>T | synonymous | 15 |
| Kafkas-SARSCoV2-020 |  |  | 23403 | S | NA | p.(Asp614Gly) | c.1841A>G | missense | 47 |
| Kafkas-SARSCoV2-020 |  |  | 25563 | ORF3a | NA | p.(Gln57His) | c.171G>T | missense | 16 |
| Kafkas-SARSCoV2-020 |  |  | 28857 | N | NA | p.(Arg195Ile) | c.584G>T | missense | 6 |
| Kafkas-SARSCoV2-021 | MT787582 | EPI_ISL_495431 | 241 | 5'UTR | NA | NA | c.1-25C>T | Non-coding | 47 |
| Kafkas-SARSCoV2-021 |  |  | 313 | ORF1ab | leader protein | p.(Leu16=) | c.48C>T | synonymous | 22 |
| Kafkas-SARSCoV2-021 |  |  | 2509 | ORF1ab | nsp2 | p.(Pro748=) | c.2244C>T | synonymous | 22 |
| Kafkas-SARSCoV2-021 |  |  | 3037 | ORF1ab | nsp3 | p.(Phe924=) | c.2772C>T | synonymous | 47 |
| Kafkas-SARSCoV2-021 |  |  | 5015 | ORF1ab | nsp3 | p.(Val1584Met) | c.4750G>A | missense | 13 |
| Kafkas-SARSCoV2-021 |  |  | 13620 | ORF1ab | RNA-dep RNA-pol | p.(Asp4452=) | c.13355C>T | synonymous | 21 |
| Kafkas-SARSCoV2-021 |  |  | 14408 | ORF1ab | RNA-dep RNA-pol | p.(Pro4715Leu) | c.14143C>T | missense | 47 |
| Kafkas-SARSCoV2-021 |  |  | 14724 | ORF1ab | RNA-dep RNA-pol | p.(Phe4820=) | c.14459C>T | synonymous | 22 |
| Kafkas-SARSCoV2-021 |  |  | 23403 | S | NA | p.(Asp614Gly) | c.1841A>G | missense | 47 |
| Kafkas-SARSCoV2-021 |  |  | 24262 | S | NA | p.(Met900Ile) | c.2700G>T | missense | 23 |
| Kafkas-SARSCoV2-021 |  |  | 27703 | ORF7a | NA | p.(Val104Phe) | c.310G>T | missense | 23 |
| Kafkas-SARSCoV2-021 |  |  | 28881 | N | NA | p.(Arg203Lys) | c.608G>A | missense | 28 |
| Kafkas-SARSCoV2-021 |  |  | 28882 | N | NA | p.(Arg203=) | c.609G>A | synonymous | 28 |
| Kafkas-SARSCoV2-021 |  |  | 28883 | N | NA | p.(Gly204Arg) | c.610G>C | missense | 28 |
| Kafkas-SARSCoV2-022 | MT787581 | EPI_ISL_495432 | 241 | 5'UTR | NA | NA | c.1-25C>T | Non-coding | 47 |
| Kafkas-SARSCoV2-022 |  |  | 3037 | ORF1ab | leader protein | p.(Phe924=) | c.2772C>T | synonymous | 22 |
| Kafkas-SARSCoV2-022 |  |  | 5301 | ORF1ab | nsp2 | p.(Ala1679Val) | c.5036C>T | missense | 22 |
| Kafkas-SARSCoV2-022 |  |  | 12809 | ORF1ab | nsp3 | p.(Leu4182Phe) | c.12544C>T | missense | 47 |
| Kafkas-SARSCoV2-022 |  |  | 14408 | ORF1ab | nsp3 | p.(Pro4715Leu) | c.14143C>T | missense | 13 |
| Kafkas-SARSCoV2-022 |  |  | 23403 | S | nsp6 | p.(Asp614Gly) | c.1841A>G | missense | 3 |
| Kafkas-SARSCoV2-022 |  |  | 28881 | N | RNA-dep RNA-pol | p.(Arg203Lys) | c.608G>A | missense | 21 |
| Kafkas-SARSCoV2-022 |  |  | 28882 | N | RNA-dep RNA-pol | p.(Arg203=) | c.609G>A | synonymous | 47 |
| Kafkas-SARSCoV2-022 |  |  | 28883 | N | RNA-dep RNA-pol | p.(Gly204Arg) | c.610G>C | missense | 22 |
| Kafkas-SARSCoV2-023 | MT786336 | EPI_ISL_495433 | 241 | 5'UTR | NA | NA | c.1-25C>T | Non-coding | 47 |
| Kafkas-SARSCoV2-023 |  |  | 313 | ORF1ab | NA | p.(Leu16=) | c.48C>T | synonymous | 23 |
| Kafkas-SARSCoV2-023 |  |  | 2509 | ORF1ab | NA | p.(Pro748=) | c.2244C>T | synonymous | 23 |
| Kafkas-SARSCoV2-023 |  |  | 3037 | ORF1ab | NA | p.(Phe924=) | c.2772C>T | synonymous | 28 |
| Kafkas-SARSCoV2-023 |  |  | 5015 | ORF1ab | NA | p.(Val1584Met) | c.4750G>A | missense | 28 |
| Kafkas-SARSCoV2-023 |  |  | 11083 | ORF1ab | NA | p.(Leu3606Phe) | c.10818G>T | missense | 28 |
| Kafkas-SARSCoV2-023 |  |  | 13620 | ORF1ab | NA | p.(Asp4452=) | c.13355C>T | synonymous | 47 |
| Kafkas-SARSCoV2-023 |  |  | 14408 | ORF1ab | nsp3 | p.(Pro4715Leu) | c.14143C>T | missense | 47 |
| Kafkas-SARSCoV2-023 |  |  | 14724 | ORF1ab | nsp3 | p.(Phe4820=) | c.14459C>T | synonymous | 1 |
| Kafkas-SARSCoV2-023 |  |  | 23403 | S | nsp9 | p.(Asp614Gly) | c.1841A>G | missense | 3 |
| Kafkas-SARSCoV2-023 |  |  | 24262 | S | RNA-dep RNA-pol | p.(Met900Ile) | c.2700G>T | missense | 47 |
| Kafkas-SARSCoV2-023 |  |  | 27703 | ORF7a | NA | p.(Val104Phe) | c.310G>T | missense | 47 |
| Kafkas-SARSCoV2-023 |  |  | 28881 | N | NA | p.(Arg203Lys) | c.608G>A | missense | 28 |
| Kafkas-SARSCoV2-023 |  |  | 28882 | N | NA | p.(Arg203=) | c.609G>A | synonymous | 28 |
| Kafkas-SARSCoV2-023 |  |  | 28883 | N | NA | p.(Gly204Arg) | c.610G>C | missense | 28 |
| Kafkas-SARSCoV2-024 | MT786337 | EPI_ISL_495434 | 241 | 5'UTR | NA | NA | c.1-25C>T | Non-coding | 47 |
| Kafkas-SARSCoV2-024 |  |  | 3037 | ORF1ab | nsp3 | p.(Phe924=) | c.2772C>T | synonymous | 47 |
| Kafkas-SARSCoV2-024 |  |  | 7765 | ORF1ab | nsp3 | p.(Ser2500=) | c.7500C>T | synonymous | 14 |
| Kafkas-SARSCoV2-024 |  |  | 8326 | ORF1ab | nsp3 | p.(Asp2687=) | c.8061C>T | synonymous | 14 |
| Kafkas-SARSCoV2-024 |  |  | 14408 | ORF1ab | RNA-dep RNA-pol | p.(Pro4715Leu) | c.14143C>T | missense | 47 |
| Kafkas-SARSCoV2-024 |  |  | 16428 | ORF1ab | helicase | p.(Tyr5388=) | c.16163C>T | synonymous | 6 |
| Kafkas-SARSCoV2-024 |  |  | 17690 | ORF1ab | helicase | p.(Ser5809Leu) | c.17425C>T | missense | 14 |
| Kafkas-SARSCoV2-024 |  |  | 18877 | ORF1ab | 3'-to-5' exonuclease | p.(Leu6205=) | c.18612C>T | synonymous | 15 |
| Kafkas-SARSCoV2-024 |  |  | 23403 | S | NA | p.(Asp614Gly) | c.1841A>G | missense | 47 |
| Kafkas-SARSCoV2-024 |  |  | 25563 | ORF3a | NA | p.(Gln57His) | c.171G>T | missense | 16 |
| Kafkas-SARSCoV2-024 |  |  | 28857 | N | NA | p.(Arg195Ile) | c.584G>T | missense | 6 |
| Kafkas-SARSCoV2-025 | MT787580 | EPI_ISL_495435 | 241 | 5'UTR | NA | NA | c.1-25C>T | Non-coding | 47 |
| Kafkas-SARSCoV2-025 |  |  | 3037 | ORF1ab | nsp3 | p.(Phe924=) | c.2772C>T | synonymous | 47 |
| Kafkas-SARSCoV2-025 |  |  | 13720 | ORF1ab | RNA-dep RNA-pol | p.(Pro4486Ser) | c.13455C>T | missense | 1 |
| Kafkas-SARSCoV2-025 |  |  | 14408 | ORF1ab | RNA-dep RNA-pol | p.(Pro4715Leu) | c.14143C>T | missense | 47 |
| Kafkas-SARSCoV2-025 |  |  | 23403 | S | NA | p.(Asp614Gly) | c.1841A>G | missense | 47 |
| Kafkas-SARSCoV2-025 |  |  | 28881 | N | NA | p.(Arg203Lys) | c.608G>A | missense | 28 |
| Kafkas-SARSCoV2-025 |  |  | 28882 | N | NA | p.(Arg203=) | c.609G>A | synonymous | 28 |
| Kafkas-SARSCoV2-025 |  |  | 28883 | N | NA | p.(Gly204Arg) | c.610G>C | missense | 28 |
| Kafkas-SARSCoV2-026 | MT789689 | EPI_ISL_495436 | 241 | 5'UTR | NA | NA | c.1-25C>T | Non-coding | 47 |
| Kafkas-SARSCoV2-026 |  |  | 313 | ORF1ab | leader protein | p.(Leu16=) | c.48C>T | synonymous | 22 |
| Kafkas-SARSCoV2-026 |  |  | 2509 | ORF1ab | nsp2 | p.(Pro748=) | c.2244C>T | synonymous | 22 |
| Kafkas-SARSCoV2-026 |  |  | 3037 | ORF1ab | nsp3 | p.(Phe924=) | c.2772C>T | synonymous | 47 |
| Kafkas-SARSCoV2-026 |  |  | 4148 | ORF1ab | nsp3 | p.(Val1295Phe) | c.3883G>T | missense | 2 |
| Kafkas-SARSCoV2-026 |  |  | 5015 | ORF1ab | nsp3 | p.(Val1584Met) | c.4750G>A | missense | 13 |
| Kafkas-SARSCoV2-026 |  |  | 13620 | ORF1ab | RNA-dep RNA-pol | p.(Asp4452=) | c.13355C>T | synonymous | 21 |
| Kafkas-SARSCoV2-026 |  |  | 14408 | ORF1ab | RNA-dep RNA-pol | p.(Pro4715Leu) | c.14143C>T | missense | 47 |
| Kafkas-SARSCoV2-026 |  |  | 14724 | ORF1ab | RNA-dep RNA-pol | p.(Phe4820=) | c.14459C>T | synonymous | 22 |
| Kafkas-SARSCoV2-026 |  |  | 23403 | S | NA | p.(Asp614Gly) | c.1841A>G | missense | 47 |
| Kafkas-SARSCoV2-026 |  |  | 24262 | S | NA | p.(Met900Ile) | c.2700G>T | missense | 23 |
| Kafkas-SARSCoV2-026 |  |  | 27703 | ORF7a | NA | p.(Val104Phe) | c.310G>T | missense | 23 |
| Kafkas-SARSCoV2-026 |  |  | 28166 | ORF8 | NA | p.(Gln91=) | c.273G>A | synonymous | 2 |
| Kafkas-SARSCoV2-026 |  |  | 28881 | N | NA | p.(Arg203Lys) | c.608G>A | missense | 28 |
| Kafkas-SARSCoV2-026 |  |  | 28882 | N | NA | p.(Arg203=) | c.609G>A | synonymous | 28 |
| Kafkas-SARSCoV2-026 |  |  | 28883 | N | NA | p.(Gly204Arg) | c.610G>C | missense | 28 |
| Kafkas-SARSCoV2-027 | MT787500 | EPI_ISL_495437 | 210 | 5'UTR | NA | NA | c.1-56G>T | Non-coding | 1 |
| Kafkas-SARSCoV2-027 |  |  | 241 | 5'UTR | NA | NA | c.1-25C>T | Non-coding | 47 |
| Kafkas-SARSCoV2-027 |  |  | 2086 | ORF1ab | nsp2 | p.(Gln607His) | c.1821G>T | missense | 1 |
| Kafkas-SARSCoV2-027 |  |  | 3037 | ORF1ab | nsp3 | p.(Phe924=) | c.2772C>T | synonymous | 47 |
| Kafkas-SARSCoV2-027 |  |  | 7765 | ORF1ab | nsp3 | p.(Ser2500=) | c.7500C>T | synonymous | 14 |
| Kafkas-SARSCoV2-027 |  |  | 8326 | ORF1ab | nsp3 | p.(Asp2687=) | c.8061C>T | synonymous | 14 |
| Kafkas-SARSCoV2-027 |  |  | 14408 | ORF1ab | RNA-dep RNA-pol | p.(Pro4715Leu) | c.14143C>T | missense | 47 |
| Kafkas-SARSCoV2-027 |  |  | 16428 | ORF1ab | helicase | p.(Tyr5388=) | c.16163C>T | synonymous | 6 |
| Kafkas-SARSCoV2-027 |  |  | 17690 | ORF1ab | helicase | p.(Ser5809Leu) | c.17425C>T | missense | 14 |
| Kafkas-SARSCoV2-027 |  |  | 18877 | ORF1ab | 3'-to-5' exonuclease | p.(Leu6205=) | c.18612C>T | synonymous | 15 |
| Kafkas-SARSCoV2-027 |  |  | 23403 | S | NA | p.(Asp614Gly) | c.1841A>G | missense | 47 |
| Kafkas-SARSCoV2-027 |  |  | 25563 | ORF3a | NA | p.(Gln57His) | c.171G>T | missense | 16 |
| Kafkas-SARSCoV2-027 |  |  | 28857 | N | NA | p.(Arg195Ile) | c.584G>T | missense | 6 |
| Kafkas-SARSCoV2-028 | MT787749 | EPI_ISL_495438 | 241 | 5'UTR | NA | NA | c.1-25C>T | Non-coding | 47 |
| Kafkas-SARSCoV2-028 |  |  | 313 | ORF1ab | leader protein | p.(Leu16=) | c.48C>T | synonymous | 22 |
| Kafkas-SARSCoV2-028 |  |  | 2509 | ORF1ab | nsp2 | p.(Pro748=) | c.2244C>T | synonymous | 22 |
| Kafkas-SARSCoV2-028 |  |  | 3037 | ORF1ab | nsp3 | p.(Phe924=) | c.2772C>T | synonymous | 47 |
| Kafkas-SARSCoV2-028 |  |  | 5015 | ORF1ab | nsp3 | p.(Val1584Met) | c.4750G>A | missense | 13 |
| Kafkas-SARSCoV2-028 |  |  | 13620 | ORF1ab | RNA-dep RNA-pol | p.(Asp4452=) | c.13355C>T | synonymous | 21 |
| Kafkas-SARSCoV2-028 |  |  | 14408 | ORF1ab | RNA-dep RNA-pol | p.(Pro4715Leu) | c.14143C>T | missense | 47 |
| Kafkas-SARSCoV2-028 |  |  | 14724 | ORF1ab | RNA-dep RNA-pol | p.(Phe4820=) | c.14459C>T | synonymous | 22 |
| Kafkas-SARSCoV2-028 |  |  | 23403 | S | NA | p.(Asp614Gly) | c.1841A>G | missense | 47 |
| Kafkas-SARSCoV2-028 |  |  | 24262 | S | NA | p.(Met900Ile) | c.2700G>T | missense | 23 |
| Kafkas-SARSCoV2-028 |  |  | 27703 | ORF7a | NA | p.(Val104Phe) | c.310G>T | missense | 23 |
| Kafkas-SARSCoV2-028 |  |  | 28881 | N | NA | p.(Arg203Lys) | c.608G>A | missense | 28 |
| Kafkas-SARSCoV2-028 |  |  | 28882 | N | NA | p.(Arg203=) | c.609G>A | synonymous | 28 |
| Kafkas-SARSCoV2-028 |  |  | 28883 | N | NA | p.(Gly204Arg) | c.610G>C | missense | 28 |
| Kafkas-SARSCoV2-029 | MT787745 | EPI_ISL_495439 | 241 | 5'UTR | NA | NA | c.1-25C>T | Non-coding | 47 |
| Kafkas-SARSCoV2-029 |  |  | 3037 | ORF1ab | nsp3 | p.(Phe924=) | c.2772C>T | synonymous | 47 |
| Kafkas-SARSCoV2-029 |  |  | 7765 | ORF1ab | nsp3 | p.(Ser2500=) | c.7500C>T | synonymous | 14 |
| Kafkas-SARSCoV2-029 |  |  | 8326 | ORF1ab | nsp3 | p.(Asp2687=) | c.8061C>T | synonymous | 14 |
| Kafkas-SARSCoV2-029 |  |  | 14408 | ORF1ab | RNA-dep RNA-pol | p.(Pro4715Leu) | c.14143C>T | missense | 47 |
| Kafkas-SARSCoV2-029 |  |  | 16428 | ORF1ab | helicase | p.(Tyr5388=) | c.16163C>T | synonymous | 6 |
| Kafkas-SARSCoV2-029 |  |  | 17690 | ORF1ab | helicase | p.(Ser5809Leu) | c.17425C>T | missense | 14 |
| Kafkas-SARSCoV2-029 |  |  | 18877 | ORF1ab | 3'-to-5' exonuclease | p.(Leu6205=) | c.18612C>T | synonymous | 15 |
| Kafkas-SARSCoV2-029 |  |  | 23403 | S | NA | p.(Asp614Gly) | c.1841A>G | missense | 47 |
| Kafkas-SARSCoV2-029 |  |  | 25563 | ORF3a | NA | p.(Gln57His) | c.171G>T | missense | 16 |
| Kafkas-SARSCoV2-029 |  |  | 28857 | N | NA | p.(Arg195Ile) | c.584G>T | missense | 6 |
| Kafkas-SARSCoV2-030 | MT787748 | EPI_ISL_495440 | 241 | 5'UTR | NA | NA | c.1-25C>T | Non-coding | 47 |
| Kafkas-SARSCoV2-030 |  |  | 3037 | ORF1ab | nsp3 | p.(Phe924=) | c.2772C>T | synonymous | 47 |
| Kafkas-SARSCoV2-030 |  |  | 14408 | ORF1ab | RNA-dep RNA-pol | p.(Pro4715Leu) | c.14143C>T | missense | 47 |
| Kafkas-SARSCoV2-030 |  |  | 23403 | S | NA | p.(Asp614Gly) | c.1841A>G | missense | 47 |
| Kafkas-SARSCoV2-031 | MT787742 | EPI_ISL_495441 | 241 | 5'UTR | NA | NA | c.1-25C>T | Non-coding | 47 |
| Kafkas-SARSCoV2-031 |  |  | 3037 | ORF1ab | nsp3 | p.(Phe924=) | c.2772C>T | synonymous | 47 |
| Kafkas-SARSCoV2-031 |  |  | 4504 | ORF1ab | nsp3 | p.(Glu1413Asp) | c.4239G>T | missense | 1 |
| Kafkas-SARSCoV2-031 |  |  | 12809 | ORF1ab | nsp9 | p.(Leu4182Phe) | c.12544C>T | missense | 3 |
| Kafkas-SARSCoV2-031 |  |  | 14408 | ORF1ab | RNA-dep RNA-pol | p.(Pro4715Leu) | c.14143C>T | missense | 47 |
| Kafkas-SARSCoV2-031 |  |  | 23403 | S | NA | p.(Asp614Gly) | c.1841A>G | missense | 47 |
| Kafkas-SARSCoV2-031 |  |  | 28881 | N | NA | p.(Arg203Lys) | c.608G>A | missense | 28 |
| Kafkas-SARSCoV2-031 |  |  | 28882 | N | NA | p.(Arg203=) | c.609G>A | synonymous | 28 |
| Kafkas-SARSCoV2-031 |  |  | 28883 | N | NA | p.(Gly204Arg) | c.610G>C | missense | 28 |
| Kafkas-SARSCoV2-031 |  |  | 29171 | N | NA | p.(His300Tyr) | c.898C>T | missense | 2 |
| Kafkas-SARSCoV2-032 | MT787486 | EPI_ISL_495442 | 241 | 5'UTR | NA | NA | c.1-25C>T | Non-coding | 47 |
| Kafkas-SARSCoV2-032 |  |  | 3037 | ORF1ab | nsp3 | p.(Phe924=) | c.2772C>T | synonymous | 47 |
| Kafkas-SARSCoV2-032 |  |  | 7765 | ORF1ab | nsp3 | p.(Ser2500=) | c.7500C>T | synonymous | 14 |
| Kafkas-SARSCoV2-032 |  |  | 8326 | ORF1ab | nsp3 | p.(Asp2687=) | c.8061C>T | synonymous | 14 |
| Kafkas-SARSCoV2-032 |  |  | 13517 | ORF1ab | RNA-dep RNA-pol | p.(Thr4418Ile) | c.13252C>T | missense | 1 |
| Kafkas-SARSCoV2-032 |  |  | 14408 | ORF1ab | RNA-dep RNA-pol | p.(Pro4715Leu) | c.14143C>T | missense | 47 |
| Kafkas-SARSCoV2-032 |  |  | 17690 | ORF1ab | helicase | p.(Ser5809Leu) | c.17425C>T | missense | 14 |
| Kafkas-SARSCoV2-032 |  |  | 18877 | ORF1ab | 3'-to-5' exonuclease | p.(Leu6205=) | c.18612C>T | synonymous | 15 |
| Kafkas-SARSCoV2-032 |  |  | 23403 | S | NA | p.(Asp614Gly) | c.1841A>G | missense | 47 |
| Kafkas-SARSCoV2-032 |  |  | 25563 | ORF3a | NA | p.(Gln57His) | c.171G>T | missense | 16 |
| Kafkas-SARSCoV2-033 | MT787553 | EPI_ISL_495443 | 241 | 5'UTR | NA | NA | c.1-25C>T | Non-coding | 47 |
| Kafkas-SARSCoV2-033 |  |  | 3037 | ORF1ab | nsp3 | p.(Phe924=) | c.2772C>T | synonymous | 47 |
| Kafkas-SARSCoV2-033 |  |  | 7765 | ORF1ab | nsp3 | p.(Ser2500=) | c.7500C>T | synonymous | 14 |
| Kafkas-SARSCoV2-033 |  |  | 8326 | ORF1ab | nsp3 | p.(Asp2687=) | c.8061C>T | synonymous | 14 |
| Kafkas-SARSCoV2-033 |  |  | 14408 | ORF1ab | RNA-dep RNA-pol | p.(Pro4715Leu) | c.14143C>T | missense | 47 |
| Kafkas-SARSCoV2-033 |  |  | 17690 | ORF1ab | helicase | p.(Ser5809Leu) | c.17425C>T | missense | 14 |
| Kafkas-SARSCoV2-033 |  |  | 18877 | ORF1ab | 3'-to-5' exonuclease | p.(Leu6205=) | c.18612C>T | synonymous | 15 |
| Kafkas-SARSCoV2-033 |  |  | 23403 | S | NA | p.(Asp614Gly) | c.1841A>G | missense | 47 |
| Kafkas-SARSCoV2-033 |  |  | 25563 | ORF3a | NA | p.(Gln57His) | c.171G>T | missense | 16 |
| Kafkas-SARSCoV2-034 | MT787473 | EPI_ISL_495444 | 241 | 5'UTR | NA | NA | c.1-25C>T | Non-coding | 47 |
| Kafkas-SARSCoV2-034 |  |  | 313 | ORF1ab | leader protein | p.(Leu16=) | c.48C>T | synonymous | 22 |
| Kafkas-SARSCoV2-034 |  |  | 2509 | ORF1ab | nsp2 | p.(Pro748=) | c.2244C>T | synonymous | 22 |
| Kafkas-SARSCoV2-034 |  |  | 3037 | ORF1ab | nsp3 | p.(Phe924=) | c.2772C>T | synonymous | 47 |
| Kafkas-SARSCoV2-034 |  |  | 13620 | ORF1ab | RNA-dep RNA-pol | p.(Asp4452=) | c.13355C>T | synonymous | 21 |
| Kafkas-SARSCoV2-034 |  |  | 14408 | ORF1ab | RNA-dep RNA-pol | p.(Pro4715Leu) | c.14143C>T | missense | 47 |
| Kafkas-SARSCoV2-034 |  |  | 14724 | ORF1ab | RNA-dep RNA-pol | p.(Phe4820=) | c.14459C>T | synonymous | 22 |
| Kafkas-SARSCoV2-034 |  |  | 23403 | S | NA | p.(Asp614Gly) | c.1841A>G | missense | 47 |
| Kafkas-SARSCoV2-034 |  |  | 24262 | S | NA | p.(Met900Ile) | c.2700G>T | missense | 23 |
| Kafkas-SARSCoV2-034 |  |  | 27703 | ORF7a | NA | p.(Val104Phe) | c.310G>T | missense | 23 |
| Kafkas-SARSCoV2-034 |  |  | 28881 | N | NA | p.(Arg203Lys) | c.608G>A | missense | 28 |
| Kafkas-SARSCoV2-034 |  |  | 28882 | N | NA | p.(Arg203=) | c.609G>A | synonymous | 28 |
| Kafkas-SARSCoV2-034 |  |  | 28883 | N | NA | p.(Gly204Arg) | c.610G>C | missense | 28 |
| Kafkas-SARSCoV2-035 | MT787747 | EPI_ISL_495445 | 241 | 5'UTR | NA | NA | c.1-25C>T | Non-coding | 47 |
| Kafkas-SARSCoV2-035 |  |  | 3037 | ORF1ab | nsp3 | p.(Phe924=) | c.2772C>T | synonymous | 47 |
| Kafkas-SARSCoV2-035 |  |  | 7765 | ORF1ab | nsp3 | p.(Ser2500=) | c.7500C>T | synonymous | 14 |
| Kafkas-SARSCoV2-035 |  |  | 8326 | ORF1ab | nsp3 | p.(Asp2687=) | c.8061C>T | synonymous | 14 |
| Kafkas-SARSCoV2-035 |  |  | 12111 | ORF1ab | nsp8 | p.(Ser3949Asn) | c.11846G>A | missense | 1 |
| Kafkas-SARSCoV2-035 |  |  | 14408 | ORF1ab | RNA-dep RNA-pol | p.(Pro4715Leu) | c.14143C>T | missense | 47 |
| Kafkas-SARSCoV2-035 |  |  | 16428 | ORF1ab | helicase | p.(Tyr5388=) | c.16163C>T | synonymous | 6 |
| Kafkas-SARSCoV2-035 |  |  | 17690 | ORF1ab | helicase | p.(Ser5809Leu) | c.17425C>T | missense | 14 |
| Kafkas-SARSCoV2-035 |  |  | 18877 | ORF1ab | 3'-to-5' exonuclease | p.(Leu6205=) | c.18612C>T | synonymous | 15 |
| Kafkas-SARSCoV2-035 |  |  | 23403 | S | NA | p.(Asp614Gly) | c.1841A>G | missense | 47 |
| Kafkas-SARSCoV2-035 |  |  | 25563 | ORF3a | NA | p.(Gln57His) | c.171G>T | missense | 16 |
| Kafkas-SARSCoV2-035 |  |  | 28857 | N | NA | p.(Arg195Ile) | c.584G>T | missense | 6 |
| Kafkas-SARSCoV2-036 | MT787743 | EPI_ISL_495446 | 241 | 5'UTR | NA | NA | c.1-25C>T | Non-coding | 47 |
| Kafkas-SARSCoV2-036 |  |  | 313 | ORF1ab | leader protein | p.(Leu16=) | c.48C>T | synonymous | 22 |
| Kafkas-SARSCoV2-036 |  |  | 2509 | ORF1ab | nsp2 | p.(Pro748=) | c.2244C>T | synonymous | 22 |
| Kafkas-SARSCoV2-036 |  |  | 3037 | ORF1ab | nsp3 | p.(Phe924=) | c.2772C>T | synonymous | 47 |
| Kafkas-SARSCoV2-036 |  |  | 5015 | ORF1ab | nsp3 | p.(Val1584Met) | c.4750G>A | missense | 13 |
| Kafkas-SARSCoV2-036 |  |  | 14408 | ORF1ab | RNA-dep RNA-pol | p.(Pro4715Leu) | c.14143C>T | missense | 47 |
| Kafkas-SARSCoV2-036 |  |  | 14724 | ORF1ab | RNA-dep RNA-pol | p.(Phe4820=) | c.14459C>T | synonymous | 22 |
| Kafkas-SARSCoV2-036 |  |  | 23403 | S | NA | p.(Asp614Gly) | c.1841A>G | missense | 47 |
| Kafkas-SARSCoV2-036 |  |  | 24262 | S | NA | p.(Met900Ile) | c.2700G>T | missense | 23 |
| Kafkas-SARSCoV2-036 |  |  | 27703 | ORF7a | NA | p.(Val104Phe) | c.310G>T | missense | 23 |
| Kafkas-SARSCoV2-036 |  |  | 28881 | N | NA | p.(Arg203Lys) | c.608G>A | missense | 28 |
| Kafkas-SARSCoV2-036 |  |  | 28882 | N | NA | p.(Arg203=) | c.609G>A | synonymous | 28 |
| Kafkas-SARSCoV2-036 |  |  | 28883 | N | NA | p.(Gly204Arg) | c.610G>C | missense | 28 |
| Kafkas-SARSCoV2-037 | MT787506 | EPI_ISL_495447 | 241 | 5'UTR | NA | NA | c.1-25C>T | Non-coding | 47 |
| Kafkas-SARSCoV2-037 |  |  | 313 | ORF1ab | leader protein | p.(Leu16=) | c.48C>T | synonymous | 22 |
| Kafkas-SARSCoV2-037 |  |  | 2509 | ORF1ab | nsp2 | p.(Pro748=) | c.2244C>T | synonymous | 22 |
| Kafkas-SARSCoV2-037 |  |  | 3037 | ORF1ab | nsp3 | p.(Phe924=) | c.2772C>T | synonymous | 47 |
| Kafkas-SARSCoV2-037 |  |  | 5015 | ORF1ab | nsp3 | p.(Val1584Met) | c.4750G>A | missense | 13 |
| Kafkas-SARSCoV2-037 |  |  | 13620 | ORF1ab | RNA-dep RNA-pol | p.(Asp4452=) | c.13355C>T | synonymous | 21 |
| Kafkas-SARSCoV2-037 |  |  | 14408 | ORF1ab | RNA-dep RNA-pol | p.(Pro4715Leu) | c.14143C>T | missense | 47 |
| Kafkas-SARSCoV2-037 |  |  | 14724 | ORF1ab | RNA-dep RNA-pol | p.(Phe4820=) | c.14459C>T | synonymous | 22 |
| Kafkas-SARSCoV2-037 |  |  | 23403 | S | NA | p.(Asp614Gly) | c.1841A>G | missense | 47 |
| Kafkas-SARSCoV2-037 |  |  | 24262 | S | NA | p.(Met900Ile) | c.2700G>T | missense | 23 |
| Kafkas-SARSCoV2-037 |  |  | 27703 | ORF7a | NA | p.(Val104Phe) | c.310G>T | missense | 23 |
| Kafkas-SARSCoV2-037 |  |  | 28881 | N | NA | p.(Arg203Lys) | c.608G>A | missense | 28 |
| Kafkas-SARSCoV2-037 |  |  | 28882 | N | NA | p.(Arg203=) | c.609G>A | synonymous | 28 |
| Kafkas-SARSCoV2-037 |  |  | 28883 | N | NA | p.(Gly204Arg) | c.610G>C | missense | 28 |
| Kafkas-SARSCoV2-038 | MT789691 | EPI_ISL_495448 | 241 | 5'UTR | NA | NA | c.1-25C>T | Non-coding | 47 |
| Kafkas-SARSCoV2-038 |  |  | 3037 | ORF1ab | nsp3 | p.(Phe924=) | c.2772C>T | synonymous | 47 |
| Kafkas-SARSCoV2-038 |  |  | 7765 | ORF1ab | nsp3 | p.(Ser2500=) | c.7500C>T | synonymous | 14 |
| Kafkas-SARSCoV2-038 |  |  | 8326 | ORF1ab | nsp3 | p.(Asp2687=) | c.8061C>T | synonymous | 14 |
| Kafkas-SARSCoV2-038 |  |  | 14408 | ORF1ab | RNA-dep RNA-pol | p.(Pro4715Leu) | c.14143C>T | missense | 47 |
| Kafkas-SARSCoV2-038 |  |  | 17690 | ORF1ab | helicase | p.(Ser5809Leu) | c.17425C>T | missense | 14 |
| Kafkas-SARSCoV2-038 |  |  | 18877 | ORF1ab | 3'-to-5' exonuclease | p.(Leu6205=) | c.18612C>T | synonymous | 15 |
| Kafkas-SARSCoV2-038 |  |  | 23403 | S | NA | p.(Asp614Gly) | c.1841A>G | missense | 47 |
| Kafkas-SARSCoV2-038 |  |  | 25563 | ORF3a | NA | p.(Gln57His) | c.171G>T | missense | 16 |
| Kafkas-SARSCoV2-039 | MT787744 | EPI_ISL_495449 | 241 | 5'UTR | NA | NA | c.1-25C>T | Non-coding | 47 |
| Kafkas-SARSCoV2-039 |  |  | 313 | ORF1ab | leader protein | p.(Leu16=) | c.48C>T | synonymous | 22 |
| Kafkas-SARSCoV2-039 |  |  | 2509 | ORF1ab | nsp2 | p.(Pro748=) | c.2244C>T | synonymous | 22 |
| Kafkas-SARSCoV2-039 |  |  | 3037 | ORF1ab | nsp3 | p.(Phe924=) | c.2772C>T | synonymous | 47 |
| Kafkas-SARSCoV2-039 |  |  | 5015 | ORF1ab | nsp3 | p.(Val1584Met) | c.4750G>A | missense | 13 |
| Kafkas-SARSCoV2-039 |  |  | 13620 | ORF1ab | RNA-dep RNA-pol | p.(Asp4452=) | c.13355C>T | synonymous | 21 |
| Kafkas-SARSCoV2-039 |  |  | 14408 | ORF1ab | RNA-dep RNA-pol | p.(Pro4715Leu) | c.14143C>T | missense | 47 |
| Kafkas-SARSCoV2-039 |  |  | 14724 | ORF1ab | RNA-dep RNA-pol | p.(Phe4820=) | c.14459C>T | synonymous | 22 |
| Kafkas-SARSCoV2-039 |  |  | 23403 | S | NA | p.(Asp614Gly) | c.1841A>G | missense | 47 |
| Kafkas-SARSCoV2-039 |  |  | 24262 | S | NA | p.(Met900Ile) | c.2700G>T | missense | 23 |
| Kafkas-SARSCoV2-039 |  |  | 27703 | ORF7a | NA | p.(Val104Phe) | c.310G>T | missense | 23 |
| Kafkas-SARSCoV2-039 |  |  | 28881 | N | NA | p.(Arg203Lys) | c.608G>A | missense | 28 |
| Kafkas-SARSCoV2-039 |  |  | 28882 | N | NA | p.(Arg203=) | c.609G>A | synonymous | 28 |
| Kafkas-SARSCoV2-039 |  |  | 28883 | N | NA | p.(Gly204Arg) | c.610G>C | missense | 28 |
| Kafkas-SARSCoV2-040 | MT787746 | EPI_ISL_495450 | 241 | 5'UTR | NA | NA | c.1-25C>T | Non-coding | 47 |
| Kafkas-SARSCoV2-040 |  |  | 313 | ORF1ab | leader protein | p.(Leu16=) | c.48C>T | synonymous | 22 |
| Kafkas-SARSCoV2-040 |  |  | 2509 | ORF1ab | nsp2 | p.(Pro748=) | c.2244C>T | synonymous | 22 |
| Kafkas-SARSCoV2-040 |  |  | 3037 | ORF1ab | nsp3 | p.(Phe924=) | c.2772C>T | synonymous | 47 |
| Kafkas-SARSCoV2-040 |  |  | 13620 | ORF1ab | RNA-dep RNA-pol | p.(Asp4452=) | c.13355C>T | synonymous | 21 |
| Kafkas-SARSCoV2-040 |  |  | 14408 | ORF1ab | RNA-dep RNA-pol | p.(Pro4715Leu) | c.14143C>T | missense | 47 |
| Kafkas-SARSCoV2-040 |  |  | 14724 | ORF1ab | RNA-dep RNA-pol | p.(Phe4820=) | c.14459C>T | synonymous | 22 |
| Kafkas-SARSCoV2-040 |  |  | 23403 | S | NA | p.(Asp614Gly) | c.1841A>G | missense | 47 |
| Kafkas-SARSCoV2-040 |  |  | 24262 | S | NA | p.(Met900Ile) | c.2700G>T | missense | 23 |
| Kafkas-SARSCoV2-040 |  |  | 27703 | ORF7a | NA | p.(Val104Phe) | c.310G>T | missense | 23 |
| Kafkas-SARSCoV2-040 |  |  | 28881 | N | NA | p.(Arg203Lys) | c.608G>A | missense | 28 |
| Kafkas-SARSCoV2-040 |  |  | 28882 | N | NA | p.(Arg203=) | c.609G>A | synonymous | 28 |
| Kafkas-SARSCoV2-040 |  |  | 28883 | N | NA | p.(Gly204Arg) | c.610G>C | missense | 28 |
| Kafkas-SARSCoV2-041 | MT789690 | EPI_ISL_495451 | 241 | 5'UTR | NA | NA | c.1-25C>T | Non-coding | 47 |
| Kafkas-SARSCoV2-041 |  |  | 2584 | ORF1ab | nsp2 | p.(Ala773=) | c.2319T>A | synonymous | 1 |
| Kafkas-SARSCoV2-041 |  |  | 3037 | ORF1ab | nsp3 | p.(Phe924=) | c.2772C>T | synonymous | 47 |
| Kafkas-SARSCoV2-041 |  |  | 5365 | ORF1ab | nsp3 | p.(Tyr1700=) | c.5100C>T | synonymous | 1 |
| Kafkas-SARSCoV2-041 |  |  | 8296 | ORF1ab | nsp3 | p.(Tyr2677=) | c.8031T>C | synonymous | 1 |
| Kafkas-SARSCoV2-041 |  |  | 12076 | ORF1ab | nsp7 | p.(Asn3937=) | c.11811C>T | synonymous | 1 |
| Kafkas-SARSCoV2-041 |  |  | 14408 | ORF1ab | RNA-dep RNA-pol | p.(Pro4715Leu) | c.14143C>T | missense | 47 |
| Kafkas-SARSCoV2-041 |  |  | 16968 | ORF1ab | helicase | p.(Glu5568Asp) | c.16703G>T | missense | 1 |
| Kafkas-SARSCoV2-041 |  |  | 18877 | ORF1ab | 3'-to-5' exonuclease | p.(Leu6205=) | c.18612C>T | synonymous | 15 |
| Kafkas-SARSCoV2-041 |  |  | 19586 | ORF1ab | 3'-to-5' exonuclease | p.(Thr6441Ile) | c.19321C>T | missense | 1 |
| Kafkas-SARSCoV2-041 |  |  | 23403 | S | NA | p.(Asp614Gly) | c.1841A>G | missense | 47 |
| Kafkas-SARSCoV2-041 |  |  | 25563 | ORF3a | NA | p.(Gln57His) | c.171G>T | missense | 16 |
| Kafkas-SARSCoV2-041 |  |  | 26681 | M | NA | p.(Phe53=) | c.159C>T | synonymous | 1 |
| Kafkas-SARSCoV2-041 |  |  | 28690 | N | NA | p.(Leu139Phe) | c.417G>T | missense | 1 |
| Kafkas-SARSCoV2-041 |  |  | 28854 | N | NA | p.(Ser194Leu) | c.581C>T | missense | 1 |
| Kafkas-SARSCoV2-042 | MT787503 | EPI_ISL_495452 | 241 | 5'UTR | NA | NA | c.1-25C>T | Non-coding | 47 |
| Kafkas-SARSCoV2-042 |  |  | 313 | ORF1ab | leader protein | p.(Leu16=) | c.48C>T | synonymous | 22 |
| Kafkas-SARSCoV2-042 |  |  | 2509 | ORF1ab | nsp2 | p.(Pro748=) | c.2244C>T | synonymous | 22 |
| Kafkas-SARSCoV2-042 |  |  | 3037 | ORF1ab | nsp3 | p.(Phe924=) | c.2772C>T | synonymous | 47 |
| Kafkas-SARSCoV2-042 |  |  | 5015 | ORF1ab | nsp3 | p.(Val1584Met) | c.4750G>A | missense | 13 |
| Kafkas-SARSCoV2-042 |  |  | 13620 | ORF1ab | RNA-dep RNA-pol | p.(Asp4452=) | c.13355C>T | synonymous | 21 |
| Kafkas-SARSCoV2-042 |  |  | 14408 | ORF1ab | RNA-dep RNA-pol | p.(Pro4715Leu) | c.14143C>T | missense | 47 |
| Kafkas-SARSCoV2-042 |  |  | 14724 | ORF1ab | RNA-dep RNA-pol | p.(Phe4820=) | c.14459C>T | synonymous | 22 |
| Kafkas-SARSCoV2-042 |  |  | 23403 | S | NA | p.(Asp614Gly) | c.1841A>G | missense | 47 |
| Kafkas-SARSCoV2-042 |  |  | 24262 | S | NA | p.(Met900Ile) | c.2700G>T | missense | 23 |
| Kafkas-SARSCoV2-042 |  |  | 27703 | ORF7a | NA | p.(Val104Phe) | c.310G>T | missense | 23 |
| Kafkas-SARSCoV2-042 |  |  | 28881 | N | NA | p.(Arg203Lys) | c.608G>A | missense | 28 |
| Kafkas-SARSCoV2-042 |  |  | 28882 | N | NA | p.(Arg203=) | c.609G>A | synonymous | 28 |
| Kafkas-SARSCoV2-042 |  |  | 28883 | N | NA | p.(Gly204Arg) | c.610G>C | missense | 28 |
| Kafkas-SARSCoV2-043 | MT789688 | EPI_ISL_495453 | 241 | 5'UTR | NA | NA | c.1-25C>T | Non-coding | 47 |
| Kafkas-SARSCoV2-043 |  |  | 313 | ORF1ab | leader protein | p.(Leu16=) | c.48C>T | synonymous | 22 |
| Kafkas-SARSCoV2-043 |  |  | 2509 | ORF1ab | nsp2 | p.(Pro748=) | c.2244C>T | synonymous | 22 |
| Kafkas-SARSCoV2-043 |  |  | 3037 | ORF1ab | nsp3 | p.(Phe924=) | c.2772C>T | synonymous | 47 |
| Kafkas-SARSCoV2-043 |  |  | 5015 | ORF1ab | nsp3 | p.(Val1584Met) | c.4750G>A | missense | 13 |
| Kafkas-SARSCoV2-043 |  |  | 13620 | ORF1ab | RNA-dep RNA-pol | p.(Asp4452=) | c.13355C>T | synonymous | 21 |
| Kafkas-SARSCoV2-043 |  |  | 14408 | ORF1ab | RNA-dep RNA-pol | p.(Pro4715Leu) | c.14143C>T | missense | 47 |
| Kafkas-SARSCoV2-043 |  |  | 14724 | ORF1ab | RNA-dep RNA-pol | p.(Phe4820=) | c.14459C>T | synonymous | 22 |
| Kafkas-SARSCoV2-043 |  |  | 23403 | S | NA | p.(Asp614Gly) | c.1841A>G | missense | 47 |
| Kafkas-SARSCoV2-043 |  |  | 24262 | S | NA | p.(Met900Ile) | c.2700G>T | missense | 23 |
| Kafkas-SARSCoV2-043 |  |  | 27703 | ORF7a | NA | p.(Val104Phe) | c.310G>T | missense | 23 |
| Kafkas-SARSCoV2-043 |  |  | 28881 | N | NA | p.(Arg203Lys) | c.608G>A | missense | 28 |
| Kafkas-SARSCoV2-043 |  |  | 28882 | N | NA | p.(Arg203=) | c.609G>A | synonymous | 28 |
| Kafkas-SARSCoV2-043 |  |  | 28883 | N | NA | p.(Gly204Arg) | c.610G>C | missense | 28 |
| Kafkas-SARSCoV2-044 | MT789692 | EPI_ISL_495454 | 241 | 5'UTR | NA | NA | c.1-25C>T | Non-coding | 47 |
| Kafkas-SARSCoV2-044 |  |  | 313 | ORF1ab | leader protein | p.(Leu16=) | c.48C>T | synonymous | 22 |
| Kafkas-SARSCoV2-044 |  |  | 2509 | ORF1ab | nsp2 | p.(Pro748=) | c.2244C>T | synonymous | 22 |
| Kafkas-SARSCoV2-044 |  |  | 3037 | ORF1ab | nsp3 | p.(Phe924=) | c.2772C>T | synonymous | 47 |
| Kafkas-SARSCoV2-044 |  |  | 13620 | ORF1ab | RNA-dep RNA-pol | p.(Asp4452=) | c.13355C>T | synonymous | 21 |
| Kafkas-SARSCoV2-044 |  |  | 14408 | ORF1ab | RNA-dep RNA-pol | p.(Pro4715Leu) | c.14143C>T | missense | 47 |
| Kafkas-SARSCoV2-044 |  |  | 14724 | ORF1ab | RNA-dep RNA-pol | p.(Phe4820=) | c.14459C>T | synonymous | 22 |
| Kafkas-SARSCoV2-044 |  |  | 23403 | S | NA | p.(Asp614Gly) | c.1841A>G | missense | 47 |
| Kafkas-SARSCoV2-044 |  |  | 24262 | S | NA | p.(Met900Ile) | c.2700G>T | missense | 23 |
| Kafkas-SARSCoV2-044 |  |  | 27703 | ORF7a | NA | p.(Val104Phe) | c.310G>T | missense | 23 |
| Kafkas-SARSCoV2-044 |  |  | 28881 | N | NA | p.(Arg203Lys) | c.608G>A | missense | 28 |
| Kafkas-SARSCoV2-044 |  |  | 28882 | N | NA | p.(Arg203=) | c.609G>A | synonymous | 28 |
| Kafkas-SARSCoV2-044 |  |  | 28883 | N | NA | p.(Gly204Arg) | c.610G>C | missense | 28 |
| Kafkas-SARSCoV2-045 | MT787558 | EPI_ISL_495455 | 241 | 5'UTR | NA | NA | c.1-25C>T | Non-coding | 47 |
| Kafkas-SARSCoV2-045 |  |  | 3037 | ORF1ab | nsp3 | p.(Phe924=) | c.2772C>T | synonymous | 47 |
| Kafkas-SARSCoV2-045 |  |  | 7765 | ORF1ab | nsp3 | p.(Ser2500=) | c.7500C>T | synonymous | 14 |
| Kafkas-SARSCoV2-045 |  |  | 8326 | ORF1ab | nsp3 | p.(Asp2687=) | c.8061C>T | synonymous | 14 |
| Kafkas-SARSCoV2-045 |  |  | 14408 | ORF1ab | RNA-dep RNA-pol | p.(Pro4715Leu) | c.14143C>T | missense | 47 |
| Kafkas-SARSCoV2-045 |  |  | 17690 | ORF1ab | helicase | p.(Ser5809Leu) | c.17425C>T | missense | 14 |
| Kafkas-SARSCoV2-045 |  |  | 18877 | ORF1ab | 3'-to-5' exonuclease | p.(Leu6205=) | c.18612C>T | synonymous | 15 |
| Kafkas-SARSCoV2-045 |  |  | 23403 | S | NA | p.(Asp614Gly) | c.1841A>G | missense | 47 |
| Kafkas-SARSCoV2-045 |  |  | 25563 | ORF3a | NA | p.(Gln57His) | c.171G>T | missense | 16 |
| Kafkas-SARSCoV2-046 | MT789694 | EPI_ISL_495456 | 189 | 5'UTR | NA | NA | c.1-77G>T | Non-coding | 1 |
| Kafkas-SARSCoV2-046 |  |  | 241 | 5'UTR | NA | NA | c.1-25C>T | Non-coding | 47 |
| Kafkas-SARSCoV2-046 |  |  | 1181 | ORF1ab | nsp2 | p.(Val306Phe) | c.916G>T | missense | 1 |
| Kafkas-SARSCoV2-046 |  |  | 2040 | ORF1ab | nsp2 | p.(Thr592Ile) | c.1775C>T | missense | 1 |
| Kafkas-SARSCoV2-046 |  |  | 3037 | ORF1ab | nsp3 | p.(Phe924=) | c.2772C>T | synonymous | 47 |
| Kafkas-SARSCoV2-046 |  |  | 4422 | ORF1ab | nsp3 | p.(Arg1386His) | c.4157G>A | missense | 1 |
| Kafkas-SARSCoV2-046 |  |  | 5017 | ORF1ab | nsp3 | p.(Val1584=) | c.4752G>A | synonymous | 1 |
| Kafkas-SARSCoV2-046 |  |  | 14408 | ORF1ab | RNA-dep RNA-pol | p.(Pro4715Leu) | c.14143C>T | missense | 47 |
| Kafkas-SARSCoV2-046 |  |  | 15738 | ORF1ab | RNA-dep RNA-pol | p.(Phe5158=) | c.15473C>T | synonymous | 1 |
| Kafkas-SARSCoV2-046 |  |  | 19839 | ORF1ab | endoRNAse | p.(Asn6525=) | c.19574T>C | synonymous | 1 |
| Kafkas-SARSCoV2-046 |  |  | 23403 | S | NA | p.(Asp614Gly) | c.1841A>G | missense | 47 |
| Kafkas-SARSCoV2-046 |  |  | 25471 | ORF3a | NA | p.(Asp27Tyr) | c.79G>T | missense | 1 |
| Kafkas-SARSCoV2-046 |  |  | 28881 | N | NA | p.(Arg203Lys) | c.608G>A | missense | 28 |
| Kafkas-SARSCoV2-046 |  |  | 28882 | N | NA | p.(Arg203=) | c.609G>A | synonymous | 28 |
| Kafkas-SARSCoV2-046 |  |  | 28883 | N | NA | p.(Gly204Arg) | c.610G>C | missense | 28 |
| Kafkas-SARSCoV2-047 | MT787487 | EPI_ISL_495457 | 241 | 5'UTR | NA | NA | c.1-25C>T | Non-coding | 47 |
| Kafkas-SARSCoV2-047 |  |  | 3037 | ORF1ab | nsp3 | p.(Phe924=) | c.2772C>T | synonymous | 47 |
| Kafkas-SARSCoV2-047 |  |  | 7765 | ORF1ab | nsp3 | p.(Ser2500=) | c.7500C>T | synonymous | 14 |
| Kafkas-SARSCoV2-047 |  |  | 8326 | ORF1ab | nsp3 | p.(Asp2687=) | c.8061C>T | synonymous | 14 |
| Kafkas-SARSCoV2-047 |  |  | 14408 | ORF1ab | RNA-dep RNA-pol | p.(Pro4715Leu) | c.14143C>T | missense | 47 |
| Kafkas-SARSCoV2-047 |  |  | 16428 | ORF1ab | helicase | p.(Tyr5388=) | c.16163C>T | synonymous | 6 |
| Kafkas-SARSCoV2-047 |  |  | 17690 | ORF1ab | helicase | p.(Ser5809Leu) | c.17425C>T | missense | 14 |
| Kafkas-SARSCoV2-047 |  |  | 18877 | ORF1ab | 3'-to-5' exonuclease | p.(Leu6205=) | c.18612C>T | synonymous | 15 |
| Kafkas-SARSCoV2-047 |  |  | 23403 | S | NA | p.(Asp614Gly) | c.1841A>G | missense | 47 |
| Kafkas-SARSCoV2-047 |  |  | 25563 | ORF3a | NA | p.(Gln57His) | c.171G>T | missense | 16 |
| Kafkas-SARSCoV2-047 |  |  | 28857 | N | NA | p.(Arg195Ile) | c.584G>T | missense | 6 |
